# Fetal bovine serum: how to leave it behind in the pursuit of more reliable science

**DOI:** 10.3389/ftox.2025.1612903

**Published:** 2025-08-08

**Authors:** Tilo Weber, Atena Malakpour-Permlid, Aline Chary, Vito D’Alessandro, Leah Haut, Sebastian Seufert, Esther Veronika Wenzel, James Hickman, Karen Bieback, Joachim Wiest, Wilhelm Gerhard Dirks, Sandra Coecke, Stina Oredsson

**Affiliations:** ^1^ Animal Welfare Academy of the German Animal Welfare Federation, Neubiberg, Germany; ^2^ Department of Health Technology, Technical University of Denmark, Lyngby, Denmark; ^3^ Department of Environmental Research and Innovation, Luxembourg Institute of Science and Technology, Belvaux, Luxembourg; ^4^ Department of Cellular, Computational and Integrative Biology, University of Trento, Trento, Italy; ^5^ Bruno Cell S.r.l, Trento, Italy; ^6^ Machine Learning Expert, Zeil am Main, Germany; ^7^ Abcalis GmbH, Braunschweig, Germany; ^8^ NanoScience Technology Center, University of Central Florida, Orlando, FL, United States; ^9^ Institute of Transfusion Medicine and Immunology, Medical Faculty Mannheim, Heidelberg University, Mannheim, Germany; ^10^ Cellasys Know-How UG, Kronburg, Germany; ^11^ Heinz-Nixdorf-Chair of Biomedical Electronics, School of Computation, Information and Technology, Technical University of Munich, TranslaTUM, Munich, Germany; ^12^ Leibniz-Institute DSMZ, German Collection of Microorganisms and Cell Cultures, Braunschweig, Germany; ^13^ European Commission, Joint Research Centre (JRC), Ispra, Italy; ^14^ Department of Biology, Lund University, Lund, Sweden

**Keywords:** animal-free *in vitro*, chemically defined media, complex *in vitro* models, cryopreservation, cultivated meat, fetal bovine serum replacement, reproducibility, xeno-free and serum-free media

## Abstract

Cell cultures form the backbone for scientific research and development, but also for clinical diagnostics and biotechnology. Supplying cells *in vitro* with growth factors, hormones, and other nutrients is achieved most often by supplementing culture media with fetal bovine serum (FBS). Despite its nearly ubiquitous use, there are major reproducibility, safety, and animal welfare issues arguing the need to replace FBS. Fortunately, numerous FBS replacements have been validated and are publicly or commercially available, making it possible to leave FBS behind. Successful serum-free, animal-component-free, and chemically defined media applications are highlighted in this review for the cultivation of stem cells and organoids, the development of organ-on-a-chip systems, the bioprinting of tissues, and the production of cultivated meat, antibodies, and vaccines, including the conduct of cytotoxicity tests and the cryopreservation of cells. Moreover, the use of fully animal-free models and methodologies is further discussed to promote their broader acceptance and adoption within the global scientific research and development community. In this regard, this review discusses novel avenues to address the scientific and practical hurdles that might limit a full transition from FBS to fully defined cell culture media and offers a brief perspective on potential future directions.

## Graphical Abstract

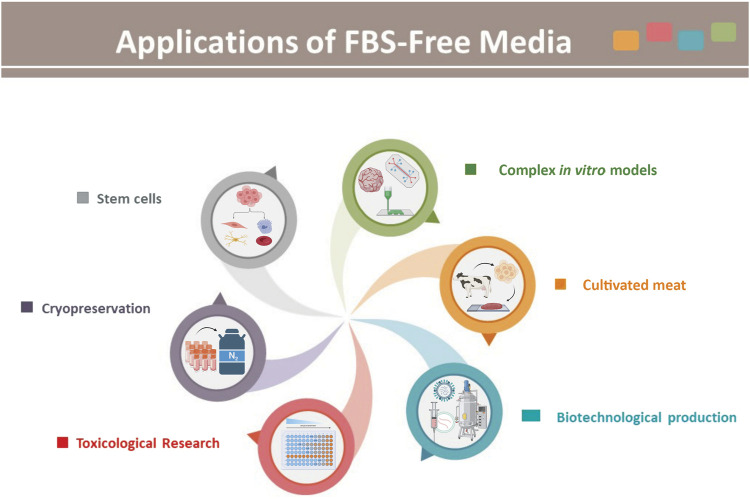

## 1 FBS-related pitfalls

In life sciences, mammalian cells are omnipresent. As the smallest structural and functional units, they are important tools for many research and development questions in biological, medical, and pharmaceutical contexts, and even in behavioral science and psychology (e.g., modeling chronic stress *in vitro*) (Heard et al., 2021). The culture of primary cells and immortalized cell lines represents fundamental achievements in biomedical research. To study living cells outside of a whole organism, it is indispensable to cultivate them in a medium that enables cellular homeostasis and mimics *in vivo* conditions.

Puck et al. (1958) kicked off the use of fetal bovine serum (FBS; also known as fetal calf serum, FCS) as a cell culture supplement in 1958, after a long and winding road of trials and errors like testing embryo juice or lymphatic fluid (Carrel, 1913; Yao and Asayama, 2017). FBS is a complex organic solution with unknown detailed composition yet rich in various amino acids, carbohydrates, hormones, lipids, proteins, vitamins, and other components that stimulate cell proliferation of adherent and suspension cells (Zheng et al., 2006; van der Valk et al., 2018). Therefore, it is widely used in cell culture media at 5%–20% concentration because vastly different cell types can pick out their specifically required substances from the highly diverse FBS composition. Since its introduction, FBS has been (nearly) everywhere: It is used in basic research, regulatory testing, production of biologicals (e.g., virus production for vaccine manufacturing), cell-based therapies, cultured meat development, toxicological testing, and cryopreservation of cells. This persistence is rather surprising, since FBS has immensely unwanted (though mostly overlooked) side effects, which have been openly discussed in the scientific community throughout the decades following its introduction (Kniazeff et al., 1967; Honn et al., 1975; Emerman et al., 1987; Kirikae et al., 1997; Jochems et al., 2002; Gstraunthaler et al., 2013; Liu et al., 2023).

### 1.1 FBS can alter model systems

Given that FBS originates from a fetal milieu, it contains various hormones and growth factors related to fetal development. These can affect and alter cells. For instance, Khodabukus and Baar (2014) engineered skeletal muscles using murine C2C12 myoblast cells and demonstrated that variations in serum origin can directly lead to changes in muscle phenotype. In another study on the healing of primary tendon tissue, van Vijven et al. (2021) showed that serum induces phenotypic drifts to such an extent that the validity of the study model can be fundamentally affected, especially in high serum concentrations. They cultured explanted mouse-tail tendon fascicles in serum-rich or serum-free medium and used transcriptome analysis to examine the altered physiology of the cells. Remarkably, a 1-week culture of tendon cells without serum showed little change in native gene expression. In contrast, FBS overrides the initial determination of primary cells concerning matrix integrity and cell morphology. Thus, this demonstrates that fetal serum-derived factors can render cell culture models unreliable. To avoid unpredictable effects of the numerous undefined serum factors in systems where precise control is required to achieve scientific aims, it is necessary to use chemically defined culture conditions from the beginning (Bieback et al., 2010). Further indication of this “rejuvenating” effect comes from a study by Kwon et al. (2016), demonstrating increased reprogramming efficacy at high serum concentrations, in which FBS modulates key molecular and cellular mechanisms. While this is a mechanism likely appreciated in this specific system, it is highly unwanted in other cell culture models, aimed to mimic physiological situations. FBS substitutes are less likely to induce unwanted reprogramming and differentiation processes (Zhao et al., 2010), even if they could initially increase the cost of media.

### 1.2 FBS can cause irreproducibility

As early as 1958 it was pointed out that the undefined and variable nature of FBS can affect the quality and performance of experiments (Puck et al., 1958; van der Valk, 2022). Variability occurs even between lots from a single manufacturer (Zheng et al., 2006; Barosova et al., 2021). Therefore, cell culture scientists, whether wittingly or unwittingly, accept a degree of uncertainty in their experimental setup, which ultimately contributes to the ongoing reproducibility crisis (Baker, 2016a; 2016b; Barosova et al., 2021; Liu et al., 2023). Nevertheless, a global survey on the use of animal-derived materials and reagents confirms that FBS is still commonly used in scientific experimentation (Cassotta et al., 2022). It is rather surprising, considering that the use of animal-derived products for scientific purposes usually requires cost-intensive pre-testing to avoid the risk of serious disruption of experiments or whole production systems by batch-to-batch variations (Bhat et al., 2021). This is mitigated by intensive pre-testing of FBS lots, to single out the ones that raise the least issues. An alternative route is using more laborious manufacturing techniques to create “purer” FBS classes suggested by Berrong et al. (2023). Either way, both will result in higher costs for the end consumer.

### 1.3 FBS can be contaminated

The use of animal-derived components always bears the risk of introducing pathogens into cell cultures. In the case of FBS, viral antibodies were first reported in 1967 (Kniazeff et al., 1967), triggering thorough examinations that confirmed the presence of viruses (Boone et al., 1971; Molander et al., 1971; Kniazeff, 1973; Kniazeff et al., 1975). Unfortunately, viral contamination of commercially available FBS remains an ongoing issue even half a century later (Paim et al., 2021; Zhang et al., 2022). Of recent concern is the spread of a pathogenic avian virus among cattle in many FBS-producing countries, causing influenza in humans with high mortality (PAHO/WHO, 2025). Moreover, various contaminants, including viruses, prions, bacteria, fungi, endotoxins, and exogenous extracellular vesicles, can be present in FBS (Kirikae et al., 1997; Reuther et al., 2006; Urzì et al., 2022). In addition, Gregersen (2008) revealed that while *Mycoplasma hyorhinis* was unable to grow in a chemically defined, serum-free medium, low amounts of serum triggered rapid growth to high titers.

Furthermore, human cells cultured in FBS can incorporate substances that are xenogenic to humans (Martin et al., 2005). This raises concerns about the safety of stem cell transplantation into humans, as it can induce an immune response in the patient, which reduces the efficacy of subsequent cell therapeutic interventions (Heiskanen et al., 2007; Sundin et al., 2007; Martin K. E. et al., 2022). Therefore, the Food and Drug Administration (FDA) of the United States of America (USA) has defined requirements for clinical-grade FBS. These include health monitoring and approval of the geographic origin of the bred animals, along with imposing minimum requirements on (current) Good Manufacturing Practice standards (cGMP or GMP), traceable certificates of origin, and testing for contamination (Minonzio and Linetsky, 2014; DiNicolas, 2015). Comparable requirements are in place in the European Union (EU) and Canada (European Commission, 2011; European Medicines Agency, 2013; Viswanathan et al., 2017). Nevertheless, these highly elaborate processes lead to increasing financial, administrative, and logistical expenses, simply to try to standardize an intrinsically unstandardized product instead of redirecting these efforts to create a standardized replacement from the beginning. Even the European Commission recommends that “when manufacturers have a choice,” the use of materials from non-animal origin is preferred (or at least materials from other species than bovines) (European Commission, 2011). And since animal-free materials suitable for cell culture media exist, manufacturers do have that choice.

### 1.4 FBS production is not transparent

The production steps of FBS are challenging to retrace (Hodgson, 1991) and a lack of transparency in the manufacturing can facilitate fraud, as scandals of serum adulteration and mislabeled geographic origin have demonstrated (Gstraunthaler et al., 2013; Gstraunthaler et al.,2014; Besser et al., 2018). Such incidents not only result in questionable experimental outcomes, but they also undermine the trust in science and industry. Subsequently, the International Serum Industry Association (ISIA) has introduced FBS traceability auditing, which has been recommended for human serum collection as well (Jacobs et al., 2023). Nonetheless, information on global annual FBS production volumes is hard to find, only estimations are publicly available (see Table 1). The most detailed numbers were published by Hodgson in 1993, stating a global supply of more than 500,000 L of raw FBS (Hodgson, 1993). In 2007, Festen estimated a worldwide production of 600,000 L per year, with half of it originating from the USA, a fifth from Australia and New Zealand, and the remainder from Canada, Central/South America, and Europe (Festen, 2007). Later information provided by the ISIA states that the USA in fact provides around 26% of the global supply (ISIA, 2020). Considering the most recent estimation from 2016, which assumed production of 200,000 L for the USA in 2017 and 2018 (RMBIO, 2016), the annual global production is projected to be close to 800,000 L (van der Valk et al., 2018). This corresponds to approximately one to two million bovine fetuses that were slaughtered in the process since the amount of obtained serum depends on the size/age of the fetuses (Jochems et al., 2002).

**TABLE 1:** Estimated global production volumes of FBS by region reported in the literature.

Region	FBS Volumes in Liters (Hodgson, 1993)	Ratio	FBS Volumes in Liters (Festen, 2007)	Ratio	FBS Volumes^a^ in Liters (RMBIO, 2016; ISIA, 2020)	Ratio^a^
Australia and New Zealand	60,000	11.5%	120,000	20.0%	154,000	20.0%
USA	210,000	40.4%	300,000	50.0%	200,000	26.0%
The Americas (without USA)	175,000^b^	33.7%	180,000	30.0%	338,000	44.0%
Europe	47,000	9.0%			50,000	6.5%
Africa	25,000	4.8%	—	—	8,000	1.0%
Others	3,000	0.6%	—	—	19,000	2.5%^c^
Grand Total	520,000^b^	100.0%	600,000	100.0%	769,000	100.0%

^a^ Numbers based on the USA volumes by RMBIO (2016) and ratios by ISIA (2020).

^b^ The original table by Hodgson (1993) displays arithmetical errors regarding the sums of these two volumes. For this table, the sums of the individual volumes of each reported country were chosen instead.

^c^ The original figure by ISIA (2020) does not display this percentage. It was calculated by subtracting the displayed percentages from 100%.

However, it must be stated that there is no official information available, not even in a supposedly highly regulated market as the EU (European Parliament, 2023), even though an official EU guidance note states that “traceability to the slaughterhouse must be assured for each batch of serum or plasma. Slaughterhouses must have available lists of farms from which the animals originate. If serum is produced from living animals, records must be available for each serum batch which assures the traceability to the farms” (European Commission, 2011). Furthermore, the European Medicines Agency insists that “the traceability of serum from final container back to the abattoir of origin is of prime importance and a clear audit trail must be demonstrable including records of volumes at each stage” (European Medicines Agency, 2013). The lack of (or will to) transparency offers latent opportunities for fraud by leaving the true production scale of FBS unknown to the public.

### 1.5 FBS can be uneconomical

Financial aspects join the ranks of disadvantages surrounding FBS. FBS prices are inherently volatile since they are interconnected with the price and supply of beef and dairy products (DiNicolas, 2015; Meat and Livestock Australia, 2016; RMBIO, 2016; Brann, 2022). Hence, if prices for these products rise (e.g., due to epizootics, natural disasters, or changing farm regulations) the price for FBS will inevitably rise as well. Trade sanctions, policies and tariffs are expected to further increase costs, especially since global tariff rates on beef were higher than those for many other agricultural-food products (Ridley et al., 2024) already before international trade tensions arose in April 2025 (Bekkers et al., 2025). In addition, beef production is expected to continuously decline over the next years in the USA, and even a relatively minor reduction by 5% could trigger a 30%–50% decrease in FBS production (Corning, 2025). This uncertainty by itself already hampers long-term cost calculations for customers and might slow down or even stop certain projects due to sudden price surges (Fife and Payne, 2025). Thus, like in every other business, rising prices prompt customers to consider reductions or replacements (Jayme, 2007; Brindley et al., 2012). In contrast, the prices of non-animal products are less susceptible to agricultural fluctuations, and easier process upscaling could further reduce costs. Therefore, it would be in the economic interest not only of customers to switch to less financially unpredictable products, but also of the serum-distributing industry to invest in animal-component-free media supplements, allowing them to diversify their business model and benefit earlier from the growing swing toward such products.

### 1.6 FBS use results in an ethical dilemma

Even though the focus of this publication is mostly on the effects of FBS on biology and medicine, the welfare of the animals involved is part of the bigger picture. Fetal calf blood is the raw material for FBS and cannot be retrieved noninvasively. As there is no scientific consensus on whether bovine fetuses suffer during and after the slaughter of the dam, the animals should be given the *benefit of the doubt* (Maurer et al., 2016; Weber et al., 2021b; McCann and Treasure, 2022). Nonetheless, even if the fetuses would be dead during blood retrieval (Versteegen et al., 2021), animal welfare issues still prevail (Weber et al., 2021b), e.g., the lack of a mandatory certified killing method including mandatory anesthesia for the fetuses (Weber and Wagner, 2021). Furthermore, pregnant dams should not be transported to slaughterhouses at all, because this inflicts additional pain, suffering, and distress on both the dams and the fetuses, potentially also resulting in premature birth or even spontaneous abortion. As an immediate step, the Eurogroup for Animals demands a transport stop for pregnant animals that have exceeded 40% of their gestation period (Porta et al., 2024).

The aspect of animal-derived materials in *in vitro* methods aiming to replace animal experiments or cultivated meat aiming to replace traditional meat production is far from being of secondary importance, as the main ethical advantages of transitioning to new techniques could be compromised if the process remains dependent on the slaughtering and suffering of animals. Therefore, FBS should be phased out to avoid this ethical dilemma.

### 1.7 FBS can be replaced

While FBS might have been a useful component in the past, there is hardly any reason for its continued use (Weber et al., 2022) as there are numerous replacement materials available in scientific literature, databases, and on the market.

Political, scientific, and economic strategies are continuously being implemented to promote the gradual phasing out of FBS to develop serum-free, defined culture media and to adapt cells to serum-free conditions (ECVAM News and Views, 2008; Knudsen and Ritskes-Hoitinga, 2021; NC3Rs, 2024). Thus, the scientific community should seek to raise awareness among cell culture researchers that FBS has been identified as a contributing factor to the reproducibility crisis and that animal-component-free replacements are available. Eventually, FBS should be limited to projects where rigorous scientific evidence demonstrates that no other viable substitute exists, rather than being used by default in routine cell cultures.

FBS provides an undefined environment for cells, and everyone should be aware that cells cultured in FBS-supplemented medium might undergo changes under its influence, resulting in an undefined cell type with unknown features and developmental potentials, which might not be physiologically representative anymore. However, every journey begins with the first step, and scientists around the globe have already gone beyond: Institutions like the “Fetal Calf Serum-free Database” make it easy to browse for existing serum replacements (van der Valk, 2021; 3Rs Centre Utrecht, 2025). This database is becoming the main place to find replacements for serum and even commercial suppliers see the giant market rising for a defined and reliable science. Meanwhile, the community of serum-free media users is growing every day, likely due to the raised awareness of its drawbacks and its role in the reproducibility crisis.

To demonstrate the feasibility of departing from FBS and establishing new and reliable gold standards for cell culture, this review presents FBS-free examples across the following fields:

• 2.1 Stem cells
• 2.2 Complex *in vitro* models
o 2.2.1. Organoids
o 2.2.2. Organ-on-a-chip systems
o 2.2.3. Bioprinting
• 2.3 Cultivated meat
• 2.4 Biotechnological production
• 2.5 Toxicological research and testing
• 2.6 Cryopreservation

In addition, strategies for adapting cells to new media are provided. To encourage researchers, financiers, and regulators to switch to FBS-free science, frequently heard statements and rebuttals on FBS use are listed in Table 2.

**TABLE 2:** Frequently heard statements and rebuttals on FBS use.

Statements (why FBS is still used)	Rebuttals (why FBS should be abolished)
It is cheaper	Prices for FBS fluctuate considerably (RMBIO, 2016), especially depending on quality. In contrast, production of animal-component-free materials will be more independent from agricultural supply chains, and upscaling will inevitably reduce their prices
It is convenient	Using FBS in cell culture includes pre-testing of FBS batches to check for their suitability, which is inconvenient and expensive at the same time. In contrast, chemically defined supplements are defined and need no laborious batch-testing
This is how it has always been done	Scientific progress lives from new inventions and ideas. Otherwise, it devolves to mere maintenance. So, do not be afraid of innovations
Never change a running system	Is the system running, though? Batch-to-batch variability can prevent it from running at all the next time (Weber et al., 2022). In addition, products used for therapeutic applications will most likely not get regulatory approval when they contain FBS.
Switching to serum-free medium prevents direct comparison with results generated under FBS conditions	Cells will behave differently when changing the culture medium. However, results in FBS-supplemented media would only be comparable when using the same FBS lot (Barosova et al., 2021), but this lot might not be available for later experiments. Thus, the possibility of comparing experimental results of cells grown in FBS-supplemented media is questionable
It is unclear how replacing FBS affects the physiology of the cells	Using FBS in the first place is in fact affecting the physiology of cells, this is just either ignored or overlooked. Serum-free cell culture media can provide a much more physiologically controlled environment
With FBS, the cells grow faster	This is another result of the unphysiological environment of serum-based medium. Slower growth might just be closer to how cells grow in the body
The test guidelines must be followed	Unless the regulated and validated processes are changed, this is unfortunately the case. However, regulators are willing to accept changes that demonstrate the same or even better results and improved reproducibility (Perez-Diaz et al., 2023; Reichstein et al., 2023)
There are no better materials available	This publication presents many better materials. In case you do not find a suitable material or strategy for you to switch to, consider consulting the following resources (Bartnicka et al., 2021; Berg and Kurreck, 2021; Duarte et al., 2023; Rafnsdóttir et al., 2023; Reichstein et al., 2023; Bramwell et al., 2024; Weber et al., 2024; 3Rs Centre Utrecht, 2025)

This review demonstrates that there is hardly any need for the continuous use of FBS anymore. FBS replacement can enhance efficiency and cost-effectiveness by improving reproducibility and thus ensuring the scientific relevance of data obtained from *in vitro* methods.

FBS can and will be fully replaced. It is not only a necessary contribution to standardize science, but an ethical necessity to ensure a transition to safer, more reproducible, and reliable research methodologies.

## 2 Replacing FBS

The use of FBS has led academia and industry into a rabbit hole from which it is hard to break out. Fortunately, innovative scientists have created viable replacements for many applications and are actively working on expanding these alternatives further. Here, several of the most prominent success stories and promising efforts on the path to FBS-free science are presented.

Supplementing media with 10% FBS is largely historical, as cell lines may maintain viability and growth at 5% (Velez et al., 1986) or lower (Sulfîanti et al., 2023). Thus, this represents a first step toward reducing FBS supplementation, but it is not sufficient to offset the aforementioned disadvantages of its use. Therefore, full replacement remains the ultimate goal.

There are various terms to describe different levels of FBS-free media, including, serum-free, xeno-free, animal-component-free, and chemically defined are the most often used ones, which can be further sub-grouped and specified. However, the term “FBS-free” can cause misunderstandings or be misleading, especially when its use varies between various vendors, manufacturers, and users. The proposed nomenclature is listed in Table 3. As Karnieli et al. (2017) stated, these categories can overlap (an animal-component-free medium is also serum-free, but not necessarily the other way around). Clear and precise definitions are essential, notably regarding proprietary formulations, since statements like “with components not directly derived from animals” and “contains no animal- or human-derived components at the primary component level” (Thermo Fisher Scientific, 2025a) can create uncertainties for customers.

**TABLE 3:** Nomenclature and description of media types including variability and animal welfare benefit potential. Adapted from van der Valk et al. (2010), Karnieli et al. (2017), and Chary et al. (2022).

Type	Description	Variability	Animal Welfare Benefit Potential
Chemically Defined (CD)	All components, their respective chemical structures, and concentrations are known	Low	High
Chemically Defined Recombinant (CDR)	Like CD, plus any components originally derived from plants, animals, or humans provided as recombinant	Low	High
Chemically Defined Purified (CDP)	Like CD, plus any components derived from plants, animals, or humans are highly purified, characterized, and quantified	Low	High (in the absence of animal-derived components)
			Low (for animal-derived components)
Animal-Component-Free (ACF)	Contain no components of animal or human origin	Low (if also CD)	High
Protein-Free (PF)	Excludes high molecular weight proteins or protein fractions but may contain peptide fractions (protein hydrolysates) derived from humans, animals, or plants	Low (if also CD)	High (in the absence of animal-derived components)
			Low (for animal-derived components)
Serum-Free (SF)	Excludes human or animal serum but may contain discrete proteins or bulk protein fractions-derived from humans, animals, or plants (e.g., human or animal tissue or plant extracts)	Low (if also CD)	High (in the absence of animal-derived components)
			Low (for animal-derived components)
Xeno-Free (XF)	Contains animal-derived components only if from the same species as the cells used. May contain components, such as animal- or human-derived growth factors	Low (if also CD)	High (if human-derived)
			Low (if animal-derived)
Serum-Based (SB)	Typically contain animal or human sera	High	High (if human-derived)
			Low (if animal-derived)

It should be noted that human-derived components can fall under the definition of animal-derived components, i.e., animal-component-free would mean that it “does not contain components of animal or human origin” (van der Valk et al., 2010; Chary et al., 2022). While this definition is not used universally (Hemeda et al., 2014), it is technically correct, as humans are obviously animals as well. Nevertheless, in the light of ethics and animal welfare, a label like “animal-free” is still helpful for products containing human-derived components. To avoid misinterpretation, it is recommended that the use of human-derived components be clearly specified.

Always bear in mind that media (supplements) with undisclosed composition/concentration are not an optimal choice for good science. The use of proprietary materials can, in itself, pose a challenge to reliability and reproducibility: When the compositions and/or concentrations are undisclosed, it becomes impossible to dissect their potential effects on cells, and there is always a risk of unnoticed composition changes due to economic factors (van der Valk, 2022). Mitsuhashi (2018) put it in a nutshell: Proprietary media “cannot be used for biochemical studies of cultured cells because the formulation has not been disclosed”. In contrast, producing laboratory materials with disclosed formulation can offer financial benefits for commercial producers, as customers can always rely on receiving the same product and are transparently informed of any modifications to the compositions.

### 2.1 Stem cells

The stem cell field is a prototype example documenting how the use of serum, in general, can impair scientific progress. The plethora of factors influencing the embryonic milieu during the bovine fetus development have been shown to prevent controlled culture and differentiation of primitive stem cells, including hematopoietic stem cells, embryonic stem cells (ESCs), and induced pluripotent stem cells (iPSCs) (Lebkowski et al., 1995; Shin et al., 2019). The undefined factors present in FBS and in other serum preparations can trigger spontaneous differentiation in the human system. Quite surprisingly, this issue has not been observed in murine ESCs (Koestenbauer et al., 2006). Thus, human ESCs and iPSCs should be cultured *per se* under chemically defined conditions, since this is the only option to ensure controlled differentiation and prevent spontaneous differentiation into unwanted lineages.

#### 2.1.1 Chemically defined media for stem cell culture

Several efforts have been made to replace serum and animal-derived products in stem cell cultures, but it is difficult to find a medium truly free of animal components. For manufacturing human iPSCs, Rivera-Ordaz et al. (2021) list several proprietary cGMP-compliant products. Nonetheless, to fully examine the biology of stem cells, a fully disclosed medium is essential. Li et al. (2023) supplemented low-glucose DMEM with different substances like human-derived proteins and recombinant growth factors to induce tenogenic differentiation and enhance proliferation of mesenchymal stem/stromal cells (MSCs). In another approach, Wu et al. (2020) developed serum-free, xeno-free, and chemically defined medium for the derivation of clinical-grade umbilical cord-derived MSCs under GMP conditions, fully replacing all serum components with synthetic/recombinant substances. Likewise, Ng et al. (2008) published a protocol to prepare their medium for the differentiation of human ESCs. Furthermore, Kuo et al. (2020) described B8, a chemically defined culture medium for human iPSCs. This medium can be prepared in-house using recombinant proteins produced in *Escherichia coli*. It supports high growth rates at low seeding densities, requires minimal medium exchange, and is cost-effective. Additionally, it maintains differentiation reproducibility and supports both the generation and long-term culture for over 100 passages (Kuo et al., 2020). Yet the reliance on Matrigel® renders it unsuitable for clinical applications (Hua et al., 2022). Matrigel® is one of the most commonly used basement membrane extracts for plate coating as well as an extracellular matrix for tissue engineering and regenerative medicine (Hughes et al., 2010; Benwood et al., 2021). However, similar to FBS, it also presents serious issues related to batch-to-batch variability, lack of reproducibility in experimental results, risk of xenogenic contamination, and severe animal welfare concerns (Aisenbrey and Murphy, 2020; Berg and Kurreck, 2021), because it is produced from Engelbreth-Holm-Swarm tumors grown in live mice (Orkin et al., 1977; Kibbey, 1994; Kleinman, 1998). Therefore, it should be replaced with human-derived, recombinant or synthetic products (Miyazaki et al., 2008; Villa-Diaz et al., 2010; Hackethal et al., 2018; Aisenbrey and Murphy, 2020; NC3Rs, 2021) listed in the “Basement Membrane Extract-free Database” (3Rs Centre Utrecht, 2025).

#### 2.1.2 Human platelet lysates for stem cell culture: Benefits and challenges

It was already in 1980 that human platelet lysate (hPL) was proposed as a replacement for FBS to culture rabbit articular chondrocytes and cell lines from a variety of tissues and tumors (Choi et al., 1980). While hPL would be xenogenic to non-human-derived cells, it serves as a truly xeno-free replacement for FBS in human-derived cell cultures, similar to human serum (Witzeneder et al., 2013; Burnouf et al., 2016; Bramwell et al., 2024; Cochrane et al., 2024). It is produced by freeze-thaw lysis of human platelets, typically obtained from expired platelet concentrate units (Hemeda et al., 2014; Fitzi-Rathgen, 2018; Mentari et al., 2022). For personalized *in vitro* medical research and testing, an autologous retrieval of platelets from the individual patient can be an option (Lang et al., 2018).

Yet platelets are by nature very heterogeneous. Thus, as for FBS, large batches of hPL are produced to minimize batch-to-batch variations and to preferably standardize reproducibility in cell-based therapeutics. Despite its potential, many challenges are still to be met (Lang et al., 2018; Bieback et al., 2019), in particular from a manufacturer’s perspective. These include ethical and economic issues related to sourcing and supply, similar to those associated with human serum (Jacobs et al., 2023). To avoid any ethical concerns over resources for blood-dependent medical applications, exclusively products past their expiry date should be used as sources for cell culture media (Weber et al., 2022; Jacobs et al., 2023; Riley et al., 2023). From a regulatory point of view, the most critical concern in utilizing blood-derived products is the potential contamination risk. This becomes even more important when pooling multiple blood donations into large hPL batch sizes, required for large-scale manufacturing of standardized product qualities (Bieback et al., 2019; Schallmoser et al., 2020). Currently, pathogen reduction technologies are evaluated to overcome this challenge.

Moreover, to prevent coagulation, hPL is usually supplemented with heparin, which is commonly of porcine or bovine origin. To avoid introducing animal-derived substances, an altered preparation method can deplete the coagulation factor fibrinogen from hPL (Kee et al., 2023), or heparin is produced using animal-free techniques (Glass, 2018; Douaisi et al., 2024). However, the major drawback remains the batch-to-batch variability and undefined nature of hPL that does not overcome reproducibility issues. In general, a chemically defined medium appears as the ideal option, because it will offer advantages for proper standardization and reproducibility.

Nevertheless, the adoption of hPL, especially in clinical-scale and GMP-compliant cell production, opened a door for improvement of MSC-based therapies (Bieback, 2013; Astori et al., 2016; Bieback et al., 2019; Tylek et al., 2019; Guiotto et al., 2020; Oeller et al., 2021). The use of hPL allows the isolation, expansion, and cryopreservation of human MSCs while maintaining their characteristics without inducing genomic instability (Trojahn Kølle et al., 2013). Some of these MSC-based therapies have immense regenerative therapeutic potential in neurological medicine (Moñivas Gallego and Zurita Castillo, 2024) and require *ex vivo* expansion and manipulation. Thus, demanding large-scale, reproducible, and high-quality cell manufacturing, which can be impaired by FBS. Despite this, a 2014 review of the FDA-based MSC regulatory filings revealed that 80% of all regulatory submissions still used FBS in human MSC manufacturing process (Mendicino et al., 2014). Similar figures were calculated when looking at human MSC-based clinical trials (Phinney and Galipeau, 2019). Since then, the impressive growth-promoting capabilities of hPL on human MSCs have been reported by numerous authors (Bieback et al., 2019; Barro et al., 2021; Nikolits et al., 2021).

Besides MSC, hPL has been shown to improve both the functionality and longevity of chimeric antigen receptor T (CAR T) cells (Canestrari et al., 2019; Torres Chavez et al., 2019). Furthermore, the therapeutic tumor-targeting effect of CAR T cells can be further enhanced via intelligent advanced media design as shown by Ghassemi et al. (2020) using a human blood-derived growth factor concentrate. Several protocols for hPL use are available, listed in more detail in specific reviews (Burnouf et al., 2016; Bieback et al., 2019; Schallmoser et al., 2020) However, particularly in the context of CAR-T and other sensitive clinical applications, the adoption of chemically defined media is preferable as a long-term goal to enhance biosafety and ensure consistent therapeutic outcomes (Immalaraju et al., 2025).

### 2.2 Complex *in vitro* models

A multitude of different two-dimensional (2D) and/or three-dimensional (3D) cell culture systems are subsumed under the term complex *in vitro* models (CIVMs). They consist of micropatterned cells, 3D microtissues (spheroids and organoids), and microphysiological systems (MPS) like organ-on-a-chip (OoC) and 3D bioprinted systems (Ekert et al., 2020; Baran et al., 2022; Parvatam et al., 2025). CIVMs provide a closer and more accurate assessment of real-life physiological conditions and enable the study of higher complex systems. Thus, external factors that influence or threaten the integrity of these systems need to be replaced with defined substances. In fact, complete independence from animal-derived materials is an important prerequisite to consider CIVMs fully fit for purpose (Weener et al., 2024).

#### 2.2.1 Organoids

Organoids are 3D multicellular structures that comprise certain levels of tissue and organ functions and they can be derived from whole organ or tumor samples, as well as from stem cells (Takebe and Wells, 2019). These miniaturized tissue models show self-organization, self-renewal, and differentiation into the functional cells of an organ (Corrò et al., 2020; Sakalem et al., 2021). Organoids are capable of wielding the primordial functions of the corresponding organ and demonstrate long-term survival (Sakalem et al., 2021). To ensure their usability and effectiveness in research and testing, organoid production must minimize unwanted heterogeneity (Louey et al., 2021). Hence, standardizing the culture materials is the foundation for achieving this task (Zhou et al., 2023). As mentioned earlier, the effects of FBS on stem cell differentiation demonstrate its use as particularly unsuitable for stem cell-derived organoids.

For optimal assembly and generation of organoids, a mechanical supportive structure is often required to assist stratification and cellular deposition. Fortunately, advances in animal-free materials made it possible for scientists like Rashidi et al. (2022), who successfully substituted FBS and Matrigel® with hPL and recombinant laminin, respectively, to generate retinal organoids. They also cultured ESCs in xeno-free Essential 8™ medium prior to the organoid formation. In addition, since FBS is commonly added to enhance the release and stability of the recombinantly produced growth factor Wnt3A, which is necessary for organoid expansion, Liefting and Bajramovic (2025) explore FBS-free methods to induce Wnt signaling in organoid expansion media, including soluble Wnt mimetics, carriers, and small molecule inhibitors.

While organoids on their own could already represent a quasi–*in vivo* modeling system (Gleave et al., 2020), further scientific possibilities emerge when combined with other 2D or 3D cell cultures into MPS as demonstrated in the following chapters.

#### 2.2.2 Organ-on-a-chip systems

Lately, OoC microfluidic systems have emerged to provide a more reliable *in vivo*-like microenvironment (Cho and Yoon, 2017), to replicate multicellular signaling cascades *in vitro* (Ortiz-Collazos et al., 2024), and to better mimic human tissue physiology where the cells are grown inside a microfluidic chip (Leung et al., 2022). OoC systems can be applied as either a single-OoC model or a complex interconnected multi-OoC system, allowing the study of individual organoid functions or systemic interactions between various organoids (Leung et al., 2022; Wilkinson, 2023; Cassotta et al., 2025). Even personalized medical research could be enabled by using patient-derived iPSCs to fabricate a “body-on-a-chip” or “you-on-a-chip” (Hayden, 2020).

Serum-free media has emerged as a crucial component in the development of less complex single-OoC models, aiming to offer more physiologically relevant and reproducible *in vitro* systems. A kidney-on-a-chip model using a hormonally defined, serum-free medium was developed to perform nephrotoxicity studies using the conventional chemotherapy drug cisplatin (Kimura et al., 2024). In another study, a muscle-on-a-chip model was developed under controlled culture conditions using a serum-free culture system, in which human skeletal myotubes demonstrated advanced differentiation and spontaneous contraction (Guo et al., 2014). Stancescu et al. (2015) established a heart-on-a-chip model derived from human ESCs using serum-free medium formulation to study the basic physiology and toxicology of human whole heart function. The data from this study demonstrated that the functional responses of *in vitro* cardiomyocytes are aligned with results derived from human clinical observations. However, these studies use serum-free media that include various animal-derived products, which should be replaced by non-animal products.

An issue concerning the generation of multi-OoC systems is the selection of cell culture media, as each organoid requires a supply of specific nutrients to promote cell adhesion, differentiation, and proliferation. Therefore, to sustain the proliferation and support of the long-term culture of several organoids, a single, optimized, and common cell culture medium is required that can support the tissue-specific function of all the organoids within a multi-OoC systems platform (Low et al., 2021; Picollet-D’hahan et al., 2021). Previous research suggests that the undefined supplement FBS should be eliminated from MPS to achieve a more faithful *in vitro* recapitulation of the functional performance of hepatocytes in the liver-on-a-chip and other interconnected multi-OoC systems (Hughes et al., 2017). Despite the description of a serum-free system, the routine culturing of cells is still performed in serum-based media. To truly earn the title of a fully serum-free system, cell culture research has to boldly go the extra mile to implement it throughout the entire cell cultivation cycle. Moreover, the addition of serum to the growth medium causes numerous significant technical adverse effects and challenges when operating microfluidics systems, including pumping difficulties, bubble formation, altered fluid dynamics, and flow rate fluctuations, particularly when serum concentrations in the medium are high (Hughes et al., 2017).

The Hickman group began with a serum-free culture system for neuronal cells in 1995 (Schaffner et al., 1995) and has now extended this from rat to mouse, both embryonic and adult, as well as to human and chimeric systems of human and rat constructs. This serum-free base medium system enables the culture of a wide range of cell types up to several months and removes a major source of variability in the system. It can satisfactorily support several neuronal, organ, endothelial and epithelial cells; see Supplementary Table S1. Furthermore, in most cases, the cells have been shown to maintain functionality for at least 1–2 months. Hence, it can provide a stable basis for connecting several different cell types simultaneously into a multi-OoC system. Nonetheless, it is important to note that these serum-free OoC systems contain animal-derived materials, which should be substituted as mentioned above.

The potential of bone marrow-derived MSCs (BMSCs) and adipose tissue-derived MSCs (ASCs) to induce and support the formation of microvessel networks and vasculogenesis in a 3D microfluidic chip platform was investigated by Mykuliak et al. (2022). Using a medium supplemented with human serum instead of the FBS, the study demonstrated that human BMSCs have a higher vasculogenic potential in the 3D microfluidic system compared to the human ASCs. In another study by Isosaari et al. (2023), BMSCs, ASCs, and endothelial cells were multi-cultured to generate 3D neurovascular networks on a chip using an open-access medium supplemented with human serum. Based on this work, the same research group later developed an open-top microfluidic chip to generate two distinct yet interconnected 3D co-culture microvascular networks with human umbilical vein endothelial cells (HUVECs) and ASCs. For both routine cell culture and the experimental setup, the researchers consistently used an endothelial cell growth medium with 2% human serum (Yrjänäinen et al., 2024). Suominen et al. (2023) successfully co-cultured iPSC-derived hepatocyte-like cells with HUVECs and ASCs for biofabrication of a functional *in vitro* liver model on a chip using medium supplemented with human serum. They have demonstrated that their multilineage 3D cell model enhances the expression of mature liver marker genes and proteins and improves their functionality compared to standard 2D cultures (Suominen et al., 2023). However, it should be noted that while the above studies utilize human serum instead of FBS, the research is not entirely humanized as normal donkey serum, animal-derived primary antibodies, secondary antibodies, and bovine-derived serum proteins were used during the immunocytochemical staining.

**FIGURE 1:**
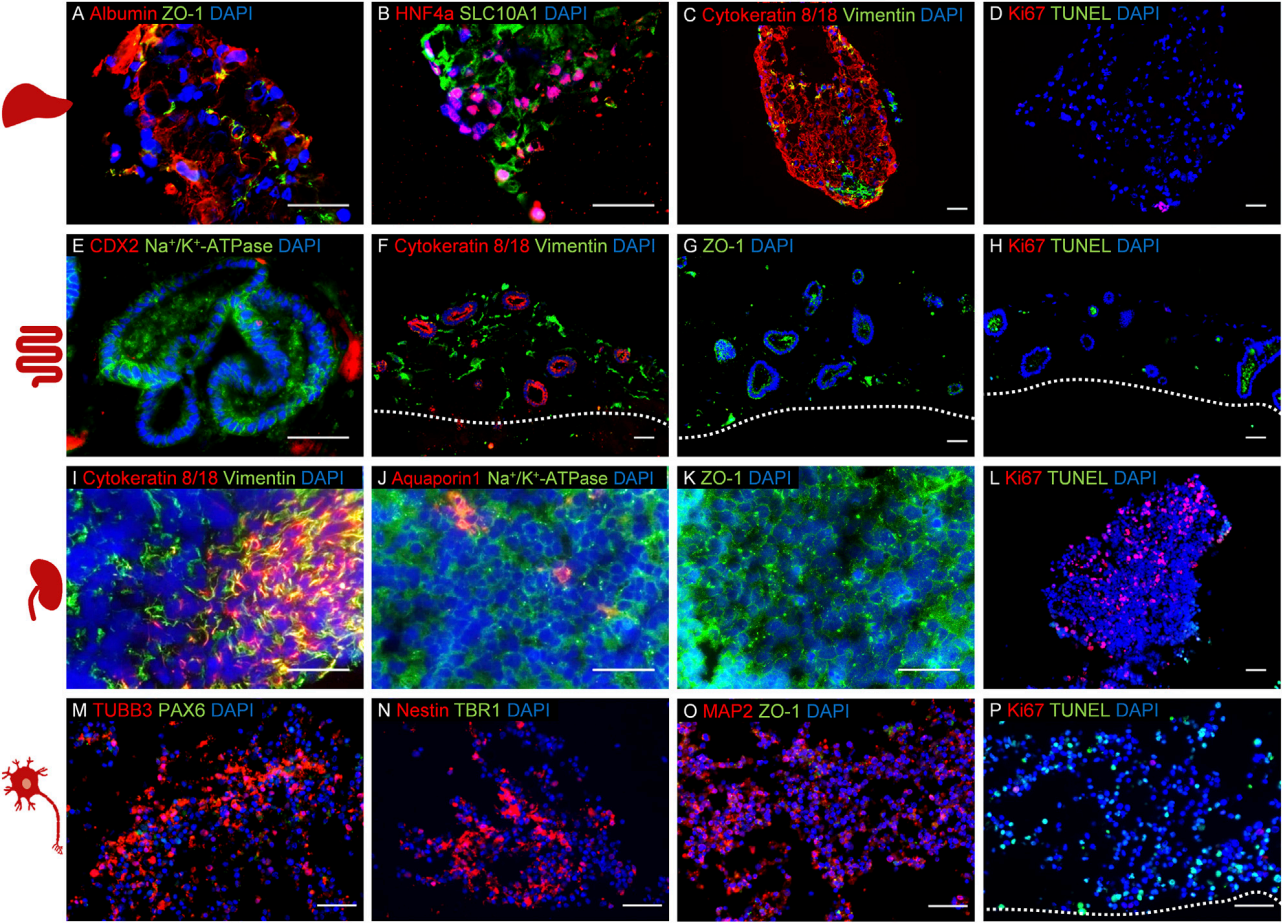
Establishment of a four organ-on-a-chip (OoC) co-culture model derived from induced pluripotent stem cells (iPSCs) using 5% human AB serum-supplemented medium over 14 days. Immunofluorescence analysis of co-cultured model representing liver **(A–D)**, intestinal **(E–H)**, renal **(I–L)**, and neuronal **(M–P)** mini-tissues. The cultures were stained to identify the following specific markers for each culture: **(A)** albumin and ZO-1, **(B)** hepatocyte nuclear factor 4 alpha and SLC10A1, **(C)** cytokeratin 8/18 and vimentin, **(D)** Ki67 and TUNEL, **(E)** CDX2 and Na+/K+-ATPase, **(F)** cytokeratin 8/18 and vimentin, **(G)** ZO-1, **(H)**: Ki67 and TUNEL, **(I)** cytokeratin 8/18 and vimentin, **(J)** aquaporin 1 and Na+/K+-ATPase, **(K)** ZO-1, **(L)** Ki67 and TUNEL, **(M)** TUBB3 and PAX6, **(N)** nestin and TBR1, **(O)** MAP2 and ZO-1, **(P)** Ki67 and TUNEL. Cell nuclei (blue) are stained in all cultures. The scale bars are 50 µm. Adapted from Ramme et al. (2019).

Multi-OoC systems have been constructed from stem cell-based systems using serum-free medium demonstrating long-term physiology (>28 days) in configurations of (for now) up to four organs (Oleaga et al., 2016). Acute and chronic compound testing in multi-OoC systems has generated drug efficacy and safety responses similar to those seen in clinical data or reports from literature (Oleaga et al., 2018). Measurement of both efficacy and toxicity has also been demonstrated in the same system for therapeutic index estimation for chemotherapeutics (McAleer et al., 2019). A recent publication described an innate immune multi-OoC system in a serum-free medium that was able to reproduce the pro-inflammatory and restorative phenotypes from macrophages (Sasserath et al., 2020) to establish a recirculating innate immune platform. In another study, a multi-channel 3D microfluidic cell culture system was developed, using a common serum-free medium supplemented with growth factors to culture four human cell types to mimic the liver, lung, kidney, and adipose tissues for drug screening applications. All four organs demonstrated well-maintained functional levels under the perfusion of serum-free medium within compartmentalized microenvironments (Zhang et al., 2009). A custom serum-free medium formulation was used to culture primary human hepatocytes and iPSC-derived cardiomyocytes with two types of cancer cells, human vulva carcinoma SW-962 and breast cancer MCF-7 cells, to construct a tumor-liver-heart-OoC system, aimed at evaluating the toxicity of anticancer drugs (McAleer et al., 2019). This study demonstrated that cultured cancer cells in the multi-OoC system supported with serum-free medium can reliably assess the impact of both conventional anticancer drugs and their metabolites *in vitro* (McAleer et al., 2019). Ramme et al. (2019) designed an autologous four OoC interconnecting miniaturized human intestine, liver, brain, and kidney using one common basic medium supplemented with 5% human AB serum as illustrated in Figure 1. They have shown that the medium was able to sustain the differentiation and phenotype of all four human iPSC-derived tissues for 14 days and the organs maintained advanced maturation with defined marker expression (Ramme et al., 2019).

Nevertheless, while medium supplemented with human serum or fully serum-free medium has been used in the experimental workflows of OoC systems, it is important to note that FBS is still used during routine cell culturing and cell maintenance in several of the studies mentioned above. Compared to the large volume of FBS-supplemented medium during routine passaging, the volume of medium required for running the OoC systems is typically small. As aforementioned, the goal should always be a full replacement of animal-derived materials throughout the entire process.

#### 2.2.3 Bioprinting

As a cutting-edge technique, 3D bioprinting uses a combination of cell sources and biological materials to construct complex functional tissues for *in vitro* disease modeling, drug screening, and clinical applications (Ma et al., 2018). 3D bioprinted tissues offer a more accurate and relevant physiological representation of human organs (Berg and Kurreck, 2021). However, even though sophisticated 3D bioprinting methods offer high accuracy and reproducibility in fabricating human tissues, they often rely on animal-derived materials, including FBS and commercially available hydrogels used as bioinks, such as Matrigel®, gelatin, and type I collagen (van der Valk et al., 2010; Martini et al., 2024). In recent years, significant efforts have been made in the field of 3D bioprinting to replace products of animal origin (Aisenbrey and Murphy, 2020; Berg and Kurreck, 2021). These novel animal-free materials are either plant-derived hydrogels, recombinant proteins, or synthetic scaffolds, which show comparable or even better biocompatibility and functionality compared to animal-derived hydrogels (Aisenbrey and Murphy, 2020). Currently, many companies have introduced plant-derived hydrogels and synthetic peptide hydrogels to support cell growth, maintain cell viability, and tissue development. Duarte et al. (2023) have extensively summarized these products in their publication. Notable examples include cellulose-based hydrogels, algae-derived alginates, agarose, and other synthetic peptide hydrogels such as BioGelx™, PeptiGels®, Synthegel® 3D Matrix and VitroGel® (Duarte et al., 2023). In addition, to fabricate humanized bioprinted organ models, human-derived extracellular matrices from donated human tissues can be decellularized and utilized as bioinks (Kabirian and Mozafari, 2020). Moreover, Santos et al. (2018), Santos et al. (2022), Santos et al. (2024) synthesized an hPL-derived porous scaffold suitable for bioprinting (Min et al., 2021). These innovations represent a significant step forward in developing more ethical and reliable materials for 3D tissue bioprinting.

By exploring serum-free media compositions and materials, researchers aim to minimize and even eradicate the need for animal-derived components for bioprinting 3D cell cultures and tissue regeneration. This is supported by the findings of Chen et al. (2022) that 3D bioprinted cancer cells are more tolerant to serum starvation conditions than cells in 2D culture. A study by Stanco et al. (2020) demonstrated the successful use of serum- and xeno-free media and cellulose/alginate bioink in bioprinting ASCs for the fabrication of vascularized tissues with high cell viability, survival, and functionality. Similarly, Lee S. H. et al. (2023) developed a serum-free bioprinted construct that supports the proliferation and spontaneous osteodifferentiation of human MSCs for osteogenesis and bone tissue regeneration. Ali et al. (2024) developed the first bioprinted liver model that is entirely free of animal-derived materials by using a chemically defined medium. They showed that 3D bioprinted liver models exhibited high cell viability (97%–101%) compared to the cell viability of a Matrigel®-based liver model (83%–102%) after 15 days of culture (Ali et al., 2024). Baltazar et al. (2023) bioprinted an implantable xeno-free vascularized human skin graft within FibroLife xeno-free complete medium, using xeno-free dermal and epidermal bioinks containing human collagen type I and fibronectin (Baltazar et al., 2023). Using a single donor of all four cell types, twelve implantable, vascularized, and bilayered skin substitutes were bioprinted under completely xeno-free culture conditions (Baltazar et al., 2023). Unfortunately, just like mentioned in the chapter on OoC systems, immunocytochemical staining was performed in this study with animal-derived sera and antibodies.

Despite this, all these studies above show that fabricating MPS through 3D bioprinting is accelerating toward animal-free conditions. Given the ongoing discussions on CIVMs/MPS standardization and improvement (CEN/CENELEC FGOoC and Secretariat: NEN, 2024; Reyes et al., 2024; Tomlinson et al., 2024), it is important to emphasize that the use of disclosed and reproducible culture media should be considered essential to these efforts.

### 2.3 Cultivated meat

The cultivation of animal cells for human or animal consumption, whether from vertebrate or invertebrate origins, represents a promising solution with potential benefits to the trade-off situation of high demand for animal protein consumption and growing animal welfare concerns (Scollan et al., 2011; Cornish et al., 2016; Kristensen, 2018; Komarek et al., 2021). Since the world’s first “lab-grown hamburger” was produced and consumed at a press conference in London in 2013 (Post, 2014), there have been significant advancements in the field. For clarity reasons, the term “cultivated meat” will be used throughout this text, even though “*in vitro* meat,” “lab-grown meat,” “cultured meat,” and “clean meat” have been used in the literature (Sexton et al., 2023; Battle et al., 2024).

Production of cultivated meat is relatively simple in its principles, involving a few essential steps (Post, 2014). Briefly, cells are collected, ideally non-invasively, which is done by isolating them from living animals maintained in optimal species-appropriate conditions. Stem cells are the most valuable for generating cell sources that can be grown for scalable production (Jara et al., 2023). Moreover, immortalized cell lines can be developed via genetic modification (Guo et al., 2025) or selected through spontaneous mutations (Ramboer et al., 2014). Following a proliferation phase, the cells are differentiated and maturated primarily into muscle and fat cells, which represent the main components of traditional meat. The mature cells are harvested to eventually undergo a post-processing phase that involves scaffolding them to provide meat-like shapes. It should be noted that although most research and development focuses on culturing bovine cells, due to the significant greenhouse gas emissions by cattle compared to other farmed animals (Gerber et al., 2013), investigations involving cells from other species are also underway (Lee D. Y. et al., 2023; Nikkhah et al., 2023; Musgrove et al., 2024; Siddiqui et al., 2024).

This section explores current and potential advancements in optimizing growth media composition for cultivated meat production, with a particular focus on achieving complete independence from animal-derived materials, which otherwise undoubtedly hinder the low-cost scale-up and commercialization of cultivated meat (Kadim et al., 2015; Stephens et al., 2018; O’Neill et al., 2021). The successful production of cultivated meat necessitates media that are safe for consumers, food-grade, cost-effective, capable of supporting large-scale cell proliferation and differentiation, possess acceptable sensory qualities, and are obviously animal-component-free (O’Neill et al., 2021).

Rather than relying on a single solution, researchers can develop serum alternatives by adopting multiple strategies, leading to the formulation of complex and chemically defined media with promising results. In this regard, Yamanaka et al. (2023) developed a novel serum-free medium containing nutrients extracted from microalga *Chlorella vulgaris* and a conditioned medium containing cell-secreted growth factors to promote the proliferation of primary bovine myoblasts. Stout et al. (2022) have taken inspiration from the aforementioned chemically defined culture medium B8 developed by Kuo et al. (2020), given its simplicity, cost-effectiveness, and capacity to positively support cell growth, and used it as a starting point for developing effective serum-free media formulations to use in cultivated meat production. Indeed, they validated a simple, serum-free, and animal-component-free medium called Beefy-9 for culturing bovine satellite cells (BSCs). This B8-inspired medium is supplemented with recombinant human serum albumin (rHSA) expressed in rice (Stout et al., 2022). Recombinant bovine serum albumin (rBSA) may serve as a future alternative in the context of species-specific cell culture (Mogilever et al., 2025). In particular, Stout et al. (2022) established a protocol for passaging BSCs in Beefy-9 and demonstrated that it maintains cell myogenicity under serum-free conditions, resulting in short-term growth comparable to that observed with 20% FBS. Further optimizations of the performance/cost ratio in Beefy-9 were achieved by adjusting the concentrations of rHSA, resulting in an improved medium called Beefy-9+. Shortly after, Kolkmann et al. (2022) developed another serum-free and chemically defined medium that effectively supported bovine myoblast proliferation at 97% efficiency compared to 20% FBS-containing growth medium. Interestingly, the albumin concentration in the medium developed by the Kolkmann group is 5 mg/mL (Kolkmann et al., 2022), which is higher compared to 0.8 mg/mL and 3.2 mg/mL in Beefy-9 and Beefy-9+, respectively (Stout et al., 2022), suggesting that rHSA significantly enhances cell proliferation and growth (Kolkmann et al., 2022). To reduce costs, Stout’s group modified Beefy-9 by replacing albumin with rapeseed protein isolate, a bulk-protein solution derived from agricultural waste streams, obtaining a new, cost-effective medium, namely Beefy-R. This new medium improved BSC growth compared to Beefy-9 while maintaining cell phenotype and mitogenicity (Stout et al., 2023). Other serum-free variants of Beefy-9, fully defined in composition and capable of in-house preparation, have been described by Schenzle et al. (2025). They tested various combinations of growth factors, myokines, and hormones, which significantly increased the proliferation rate of BSCs. Especially, the use of methylcellulose and racemic alanine, two inexpensive, food-grade stabilizers, either alone or in combination, has been proposed to efficiently enhance the propagation of muscle cells cultured in both B8 and B9 media while keeping overall production costs low. In this regard, it has to be noted that the ongoing research in microbial cell factories, particularly the optimization of *Escherichia coli* strains for recombinant growth factor and serum protein production, as well as that of purification processes, offer new opportunities in the field, not only from a cost-effectiveness perspective but also in terms of scalability, as highlighted in a recently published review (Mainali et al., 2025). Further attempts to reduce costs came from the work published by Pasitka et al. (2024), who developed an optimized animal-component-free medium where they replaced albumin with methylcellulose and hydroxypropyl β-cyclodextrin, reducing medium costs by 38% (Pasitka et al., 2024). Notably, this medium supported high-density chicken cell cultures, achieving 28 million cells/mL and enabling multiple harvests over 20 days, performing comparably to albumin-containing serum-free media, thus offering a cost-effective and scalable option for cultivated meat production (Pasitka et al., 2024). Interestingly, in 2022, Mosa Meat, a leading food technology company in the Netherlands focused on cultivated beef, developed a chemically defined, serum-free medium that induced myogenic differentiation of 3D muscle organoids, mimicking serum starvation without resorting to transgene expression (Messmer et al., 2022). As the authors themselves highlighted, the hydrogels in the study were not entirely animal-component-free, but they expect that these findings can be reproduced in fully animal-free organoid cultures. In addition, Mosa Meat researchers developed a defined serum-free medium for cultivating beef fat *in vitro* (Mitić et al., 2023), which is a key ingredient to the flavor of traditional meat. They reported superior adipogenesis in bovine stromal vascular cells differentiated in this serum-free medium compared to those in the FBS-containing one, both in 2D and 3D cultures. These findings have collectively contributed to the Dutch company’s submission of its first application for EU market approval of cultivated beef fat in 2025 (Team IO, 2025). It is important to note that, as reported previously throughout this section, not all serum-free media are necessarily animal-component-free. This distinction is crucial since the inclusion of any animal-derived ingredients in cultivated meat products would most likely discourage consumers who do not follow animal-based diets (Jaiswal and Shrivastava, 2024). Therefore, researchers are encouraged to explore alternatives to animal sera from sources outside the animal kingdom to ensure that cultivated meat production is sustainable and genuinely free of animal suffering.

Ultimately, while this discussion has primarily focused on cultivated meat production and the associated issues with FBS and animal sera, and potential animal-free supplements, the landscape of cellular agriculture extends far beyond meat. This includes cell-based milk, egg proteins, and leather, each presenting unique challenges and opportunities that are both exclusive or shared with those of cultivated meat (Eibl et al., 2021; Fytsilis et al., 2024).

Despite the progress made, further technical challenges remain, including identifying the best cell source and achieving scalability (Stephens et al., 2018). Nevertheless, knowledge in the field of cultivated animal products is advancing rapidly. One approach that includes machine learning is described in Nikkhah et al. (2023). The authors use a unique approach of several techniques to optimize culture media formulation for growing cultivated meat using a cell line from zebrafish (*Danio rerio*). To this end, a multistep approach is used that includes radial base functions with genetic algorithms to determine the problem of the optimal base media for serum creation, while the overall design is modeled with the response surface method. The use of generative approaches in general for linear and nonlinear optimization has been proven to be effective in certain aspects of bioinformatics (Li et al., 2020), and thus also promises potential to be harnessed in this context. While the authors claim a good predictability, the standard pitfalls of machine learning approaches such as overfitting or bad sample bases need to be considered.

This demonstrates that not only did cellular agriculture profit immensely from the progress published by biomedical science (Heine et al., 2024), but it can *vice versa* inspire the development of animal-free media for other scientific fields. While competition and proprietary reasons might seem to speak against publishing results obtained by cellular agriculture companies on animal-component-free cell culture media, transparency on their ingredients will boost consumer trust in the nutritional value and safety of their products.

### 2.4 Biotechnological production

In addition to cell culturing, animals have long played a role in the generation of biotechnological products, including antibodies and vaccines. These animal-derived materials were crucial for various medical and scientific applications ranging from diagnostic tools to disease prevention. Nevertheless, past reliance on animals does not justify the continued use of animals as “means of production” in perpetuity. By examining antibodies and vaccines as two prominent examples, this chapter explores the ongoing transition to animal-free production methods and highlights the technological advances and their impact on sustainability and innovation.

Recombinant antibody production is the cornerstone of modern antibody production and enables the production of monoclonal antibodies without relying on animal-intensive hybridoma technology. In this approach, antibody-encoding genes are cloned into expression vectors, which are then introduced into eukaryotic host cells such as Chinese hamster ovary (CHO) or human embryonic kidney 293 (HEK293) cells.

The sequences of the antibody genes can be obtained animal-free using display methods (McCafferty et al., 1990; Barbas et al., 1991; Breitling et al., 1991; Boder and Wittrup, 1997; Ho et al., 2006; Akamatsu et al., 2007), by polymerase chain reaction (Larrick et al., 1989; Breitling and Dübel, 1997), or next-generation sequencing of hybridoma clones (Subas Satish et al., 2022; Mitchell et al., 2023). Recombinant production systems are highly scalable, compatible with regulatory requirements, and produce antibodies with consistent quality, rendering them indispensable for therapeutic, diagnostic, and research applications.

In the production of recombinant antibodies for the pharmaceutical industry, but also diagnostics and research, it is essential to avoid potentially harmful or unknown substances during the entire production cycle. Production should be defined, scalable, and batch consistent. These criteria can be achieved through animal-free, recombinant production without FBS or other animal-derived materials. Consequently, the EU Reference Laboratory for Alternatives to Animal Testing of the Joint Research Center stated in 2020 that “non-animal-derived antibodies are well-defined and better reagents that will improve the reproducibility and relevance of scientific procedures and lead to more efficient and effective use of research funds,” while recommending that “EU countries should no longer authorise the development and production of antibodies through animal immunisation, where robust, legitimate scientific justification is lacking” (Barroso et al., 2020). This gave impetus to ongoing research into the development of animal-free antibodies for diagnostics and research (Dübel, 2024; Groff et al., 2024).

CHO and HEK293 cell lines are commonly used to produce antibodies that match the human glycosylation pattern and post-translational modification. These cell lines are optimized for bioreactors and in most cases already adapted to FBS-free medium. If not, they can be gradually adapted to FBS-free medium via out-dilution of FBS (Weber et al., 2022). CHO cells are considered the gold standard for large-scale therapeutic antibody production due to their high adaptability to serum-free and suspension culture systems. They are robust and scalable for industrial production (Reinhart et al., 2019), and despite not being of human origin, produce antibodies with human-like glycosylation patterns. Furthermore, they are suitable for most FDA-approved therapeutic monoclonal antibodies with more than 34 biosimilars produced in CHO receiving FDA approval since 2015 (Gupta et al., 2023). Alternatively, HEK293 cells are ideal for rapid, transient expression of recombinant antibodies. Since they are a human-derived cell line, this indubitably ensures human-like glycosylation. HEK293 cells are often used in early-stage research or preclinical development (Backliwal et al., 2008; Jäger et al., 2013). Several commercially available media for protein expression in cells are free of animal components and do not require FBS as an additive (Reinhart et al., 2015). A selection of commercially available media suitable for CHO and HEK293 suspension cells can be found in Table 4. It has to be noted that Cervera et al. (2011) developed a supplement consisting of recombinant proteins and an animal-component-free lipid mix, which improves HEK 293 cell density grown in Freestyle™ 293.

**TABLE 4:** Selection of commercially available, animal-component-free media with a proprietary formulation for CHO and HEK293 suspension cells.

Media Name	Cell Lines	References
4Cell^®^ SmartCHO; 4Cell^®^ XtraCHO	CHO	Wynne et al. (2025); Reger et al. (2023)
ActiCHO	CHO	Reinhart et al. (2019); Fan et al. (2018)
BalanCD CHO	CHO	Schwarz et al. (2020)
CD CHO	CHO	Reinhart et al. (2019); Fan et al. (2018); Cain et al. (2013)
FortiCHO	CHO	Xu et al. (2019)
HyClone CDM4CHO	CHO	Rodrigues et al. (2012); Paredes et al. (2013)
PowerCHO-2 CD	CHO	Fan et al. (2018)
BalanCD HEK293	HEK293	L’Abbé et al. (2018); Schwarz et al. (2020)
Expi293 expression medium	HEK293	Fang et al. (2017), Vazquez-Lombardi et al. (2018)
FreeStyle™ 293; F17 expression medium	HEK293	Wenzel et al. (2020); Jang et al. (2022); Kim et al. (2022)
HEK TF, HEK FS_2	HEK293	Marchetti (2022); Schneider et al. (2021)

Advances in cell culture technologies are rapidly expanding the capabilities of non-mammalian-based cell lines e.g., insect cells (Korn et al., 2020) and even plant cells (Donini and Marusic, 2019; Schillberg and Spiegel, 2022). Thus, it is essential to anticipate and explore innovative advancements in cell line development that could potentially revolutionize research methodologies. These advancements offer many advantages for the future, especially in research and diagnostic applications that are not dependent on human post-translational modification and glycosylation patterns.

Another prominent biotechnological application, besides recombinant antibody production, is the development of vaccines using immortalized cell lines, especially mammalian kidney-derived cells like HEK293, Madin-Darby canine kidney (MDCK), or Vero cells, but also insect-derived cells like *Spodoptera frugiperda* 9 (Sf9). Especially suspension cell cultures in combination with an animal-component-free medium are more appropriate for large-scale bioreactor-based production by creating a steadier and more controlled vaccine manufacturing process (Zhang et al., 2023). Fortunately, several animal-component-free media are available. Gélinas et al. (2019) utilized the transfection medium HyClone HyCell TransFx-H to express a promising Ebola virus vaccine in HEK293 cells. The 4Cell® MDCK CD medium is suitable for MDCK cells and also protein-free (Chaabane et al., 2021; Zinnecker et al., 2024). Chen A. et al. (2011) tested five commercially available animal-component-free media for the influenza vaccine production in Vero cells, with EX-CELL® Vero SFM achieving the highest cell concentration. Moreover, several media suitable for culturing insect cells are on the market as well (Chan and Reid, 2016; Cox, 2021). These and further examples are summarized in Table 5.

**TABLE 5:** Selection of commercially available, animal-component-free media with a proprietary formulation for cells used in vaccine production (and in other applications).

Media Name	Cell Lines	References
HyClone HyCell TransFx-H	HEK293	Gélinas et al. (2019)
4Cell^®^ MDCK CD	MDCK	Chaabane et al. (2021); Zinnecker et al. (2024)
OptiPRO™ SFM	MDCK, Vero, (BHK-21, COS-7, HeLa, MDBK, PK-15)	Nelson (2016); Lee et al. (2020)
VP-SFM	MDCK, Vero, (BHK-21, COS-7, HEp2)	Fang et al. (2021); Fusi and Dotti (2021)
4Cell^®^ NutriVero™ Flex 10	Vero	Brill et al. (2020)
EX-CELL^®^ Vero SFM	Vero	Chen et al. (2011a)
4Cell^®^ Insect CD	Sf9	Cox (2021)
ExpiSf CD	Sf9	Cox (2021)
HyClone SFM4Insect	Sf9, (Sf21, Tn5)	Chan and Reid (2016)
Sf-900™ III SFM	Sf9, (Sf21)	Chan and Reid (2016)

In addition to the commercially available media mentioned in Tables 4, 5, several disclosed formulations are also available: Schneider (1989) described the preparation of a basal medium for CHO cells as early as 1989, while Burteau et al. (2003) modified it with plant peptones to improve cultivation and productivity. More recently, Balabashin et al. (2020) described a CHO medium consisting of recombinant protein supplements and hydrolysates of non-animal origin.

For HEK293 (Oredsson et al., 2025), Vero (Rourou et al., 2009; Kuncorojakti et al., 2024; Oredsson et al., 2025), MDCK (Mochizuki, 2006), and Sf9 cells (Inlow et al., 1989; Boegel, 2019), animal-component-free media formulations can be found in the literature, which all contain proteins either from soy, yeast, human, or recombinant sources. In contrast, Wilkie et al. (1980) developed a chemically defined, protein-free medium for insect cell lines, with the detailed composition reprinted by Mitsuhashi (2018). Generally, protein-free media are recommended for cell culture designated for vaccine production to reduce extraneous antigens and avoid an unstable amino acid composition in the medium due to protein degradation (Wallis et al., 1969; Mitsuhashi, 2018). A white paper from Eppendorf consequently declares that the “holy grail” in vaccine production “being an economical, protein-free, serum-free medium that would provide strong growth support and have the property of scalability to large volumes, up to thousands of Liters, while coming in at an affordable price” (Sha, 2021). For completeness, the phrase “with a disclosed formulation” should be added to this list.

Despite the fact that FBS-supplemented medium has none of these traits, recent studies continue to use it for the cultivation of the cell lines mentioned above (Bermúdez-Abreut et al., 2025; Koide et al., 2025; Li et al., 2025; Yan et al., 2025; Yang et al., 2025). Even when cell culture collections display that a specific cell line like the conveniently named “Vero (AC-free)” is adapted to grow in animal-component-free medium (González Hernández and Fischer, 2007; UKHSA Culture Collections, 2025), one can still find publications using FBS-supplemented media instead (Riepler et al., 2020; Malekshahi et al., 2021). One cannot and should not blame the scientists for doing this, because supplementation with FBS has permeated deep into the general workflow of cell culturing. This shows the ongoing importance of disseminating information on replacing animal-derived materials, although replacements have been described before in the literature.

### 2.5 Toxicological research and testing

Toxicologists investigate the potentially harmful properties of substances, where it is essential to maintain strict control over experimental conditions. The introduction of unknown or potentially adverse components into research and test samples could significantly compromise the reliability and accuracy of findings. Therefore, it is imperative that cell culture media used in these studies are chemically defined and reproducible to facilitate good toxicological science (Rabbit et al., 2025). This enables toxicologists to isolate and study the specific effects of the research or test substances under investigation. By eliminating variability introduced by undefined components, such as those found in complex products like FBS, researchers can more accurately assess the true impact of substances on biological systems. The use of chemically defined media with limited batch variation of the components should enhance the reproducibility of experimental results aligning with principles of scientific accuracy and ethical considerations. It supports the pursuit of reliable data that can inform regulatory decisions regarding the safety and toxicity of chemicals, pharmaceuticals, and other substances. Ultimately, by applying chemically defined media standards, toxicologists can advance their understanding of potential hazards and contribute to improved public health and environmental safety practices.

A pivotal stride was a 2021 report leading major efforts to validate 17 *in vitro* mechanistic methods focused on thyroid hormone disruption and providing examples for replacing animal-derived materials in these methods (Bartnicka et al., 2021). Furthermore, an industry-sponsored initiative, launched in September 2020, aims to transition the test guidelines (TG) of the Organisation for Economic Co-operation and Development (OECD) toward animal-component-free assays (NC3Rs, 2020). This initiative seeks to enhance the reliability, reproducibility, and consistency of OECD TG by promoting the adoption of chemically defined media wherever possible. Reichstein et al. (2023) exemplified this by successfully demonstrating the replacement of animal-derived components in OECD TG 455 (estrogenic activity) and OECD TG 487 (genotoxicity) (OECD, 2021b; OECD, 2023). Similarly, Perez-Diaz et al. (2023) adapted TK6 cells to an animal product-free, chemically defined culture medium for genotoxicity studies under OECD TG 487. Other groups have also made significant strides in advancing research and testing methodologies within the field of toxicology. For instance, by refining procedures in accordance with the International Organization for Standardization (ISO) standard ISO 10993-5, which addresses *in vitro* cytotoxicity for the biological evaluation of medical devices. The methodology employs either L929 or CaCo-2 cells combined with a straightforward medium composed of a DMEM/F12 mixture supplemented with insulin-transferrin-selenium (Wiest, 2017; Weber et al., 2021a). A recent study is also focused on enhancing the reliability of toxicological assessments by developing a specialized culture medium for the long-term, serum-free cultivation of fish cells (Jožef et al., 2025). This medium is tailored specifically for a cell line assay using RTgill-W1 cells derived from the rainbow trout (*Oncorhynchus mykiss*). This assay is utilized in OECD TG 249, a standard protocol for assessing fish acute toxicity (OECD, 2021a). However, full replacement of animal-derived materials was not yet achieved in that study, but protein-free formulations supplemented with dipeptides were suggested as a viable alternative (Jožef et al., 2025). There are also studies on fully humanizing toxicological *in vitro* test systems. Fraser et al. (2025) used hPL for the toxicity testing strategy of a nanomaterial while Ward et al. (2025) presented the AcutoX method for predicting acute oral toxicity with human fibroblasts cultured in the presence of pooled human serum.

These advancements are paving the way for a future where toxicological research and testing become more reliable based on defined methodologies. By refining culture media and testing methodologies, researchers aim to enhance the reliability and accuracy of toxicological data. This, in turn, contributes to more robust, improved safety assessments and regulatory decision-making processes.

### 2.6 Cryopreservation

Not only commercial cell banks such as the American Type Culture Collection and the European Collection of Cell Cultures, but everyone who works with cell cultures, must freeze cells sooner or later, coming along with the question of how to properly thaw them to ensure successful revival. As cell lines may exhibit genetic drift after a certain number of passages, frozen cells are a useful source to refresh the cell cultures. In addition, cell lines can be accidentally lost due to infection, cross-contamination, or for various other reasons. Thus, cryopreserved master and working cell banks ensure the constant availability of cells. Proper freezing and thawing techniques are economically beneficial for each laboratory because it avoids the need to purchase new cell ampoules from vendors. Cryopreservation is used for fertility cell preservation (and future *in vitro* fertilization), stem cell and iPSCs preservation, as well as preservation of other cell types dedicated to research and clinical applications (Jaiswal and Vagga, 2022). Importantly, a standardized protocol is crucial for reproducible freezing, allowing different personnel to achieve consistent results.

Directly freezing cells in a medium with water and no cryoprotectant will be fatal. When a solution with a high concentration of water freezes, the water molecules will form ice crystals through hydrogen bonding resulting in increased salt concentration in the remaining solution. Ice crystals damage cell membranes and the elevated solute concentration causes cellular dehydration resulting in osmotic shock (Jang et al., 2017). Therefore, cryoprotectants that penetrate the cell membranes reduce ice formation are added to the cell freezing medium, and specific freezing protocols are applied. Often cryoprotectants like glycerol, dimethyl sulfoxide (DMSO), ethylene glycol, and propylene glycol are used, in combination with FBS (Whaley et al., 2021). Due to its high protein content, FBS in the freezing medium provides a certain cryoprotective effect, and concentrations up to 95% are applied (Fujisawa et al., 2019).

Currently, several cryoprotective media have been developed that dispense with FBS for obvious safety reasons. Some of these media are publicly available and are based on human serum, human blood components (Hreinsson, 2003; Reuther et al., 2006; Park et al., 2018; Rafnsdóttir et al., 2023), or combinations of defined animal-component-free cryoprotectants (González Hernández and Fischer, 2007; Volbers et al., 2016; Hsieh et al., 2018; Pless-Petig and Rauen, 2018).

Several authors have demonstrated the suitability of various proprietary commercially available FBS-free cryopreservation media (Miki et al., 2016; Chary et al., 2022; Uhrig et al., 2022). However, as mentioned previously, if the composition is proprietary, the influence of the media components on the cells cannot be investigated and therefore, it must be thoroughly studied and proven by the vendors. Thus, disclosed media formulations are preferred.

The simplest cryopreservation strategy involves using high protein concentrations in any xeno-free medium. In this regard, Hreinsson (2003) used human serum albumin (HSA) at concentrations of 25 mg/mL in phosphate-buffered salt (PBS) solution to successfully cryopreserve follicles in ovarian cortical tissue. Oredsson et al. (2025) demonstrated that cryopreservation in PBS with 20 mg/mL HSA works effectively with 5% DMSO as a cryoprotectant. CaCo-2 cells and HEK293 cells adapted to a chemically defined medium were successfully frozen in this medium to which 7.5%–10% DMSO was added (J. Wiest and E. V. Wenzel, personal communication, 3 March 2025). Therefore, using the chemically defined medium in which the cells are cultured and adding DMSO to it for freezing can be easily investigated by the user.

Pakhomov et al. (2022), Pakhomov et al. (2024a), Pakhomov et al. (2024b) reported an optimal freezing medium for testis interstitial cells consisting of 100 mg/mL Dextran 40 with 0.7 M concentration of the permeating cryoprotectant Me_2_SO in Ham’s F12 medium. This freezing medium was superior to FBS-supplemented medium. Interestingly, they investigated physical processes such as ice crystal formation in various freezing media by differential scanning calorimetry and thermomechanical analysis.

The final evidence for the suitability of the freezing method and medium as well as storage conditions is the viability of cells post-thawing and seeding. This has been investigated in studies by several authors regarding proprietary xeno-free freezing media (Miki et al., 2016; Uhrig et al., 2022) and disclosed media (Hreinsson, 2003).

## 3 Transition strategies to switch from FBS to FBS-free

Successful transition of cells to thrive and survive in new media is not trivial, often it is complex, time-consuming, or associated with higher initial costs. While some cells tolerate a sudden and complete change from one media to another (Weber et al., 2021a; Oredsson et al., 2025), others are more sensitive and need time and/or additional treatment while transitioning (Mochizuki, 2006; Jang et al., 2022). In such cases, stepwise adaptation to a new medium is the key to success. Researchers need to keep in mind that, after complete transitioning to FBS-free conditions, close monitoring of the cells is crucial to ensure consistent cell proliferation and stable cell numbers during each routine culturing over several passages (Malakpour-Permlid et al., 2025b).

The common key elements for a successful transition to a new medium are 1) a gradual adaptation to FBS-free, 2) careful monitoring of cell line functionality after the transition phase, and 3) reliable cryopreservation of cells.

Different protocols have been described in the literature such as van der Valk et al. (2010), Chary (2023), and Marigliani et al. (2019), or on FBS-free medium suppliers’ websites, including:

- Reduction in serum content: Cells are grown in the specific FBS-free medium supplemented with FBS (i.e., the FBS concentration recommended by the supplier of the cells, usually 10%), with the FBS concentration reduced at each passage until it reaches 0%.
- Sequential adaptation: Cells are cultured in a mixture of FBS-containing medium (e.g., basal medium + 10% FBS) and specific FBS-free medium, gradually increasing the proportion of FBS-free medium until the transition is complete and it reaches 100%.
- Adaptation with a conditioned medium: Similar to the sequential adaptation, cells are passaged in a mixture of media, using the medium from the previous passage.
- Shock or rapid adaptation: Cells are directly adapted to serum-free media by a direct and abrupt switch from FBS-medium to serum-free medium.

Once the cells are transitioned to an FBS-free medium, cells should be maintained in culture for three to five passages in FBS-free medium to ensure stable growth and high viability. To accelerate the prospect of developing and testing of a replacement media’s suitability, real-time assays like the cellasys #8 can be deployed (Eggert et al., 2022; Eggert et al., 2023; Wiest, 2022).

The acute myeloid leukemia monocytic THP-1 cell line (Tsuchiya et al., 1980), used as a model of dendritic cells in the human cell line activation test (h-CLAT), represents one of the validated *in vitro* models adopted by the OECD to replace *in vivo* testing for identifying skin sensitizers (Ashikaga et al., 2006; OECD, 2024). A study by Marigliani et al. (2019) aimed to replace FBS in the culture of THP-1 cells in the h-CLAT with various commercially available FBS-free media. After gradual adaptation to FBS-free media, THP-1 cells correctly predicted the sensitizing potential of ten proficiency substances (Marigliani et al., 2019). It is thus possible to eliminate the use of FBS in validated protocols, using FBS-free media. Edwards et al. (2018) also demonstrated successful replacement of FBS in the h-CLAT with human serum and beyond also replaced animal-derived antibodies with non-animal-derived antibodies (Edwards et al., 2018; Barroso et al., 2020).

Similarly, the human lung carcinoma cell line A549 (Lieber et al., 1976), a model of alveolar type II epithelial cells, which is one of the most common cell lines used in respiratory research, was successfully transitioned to two different commercially available FBS-free media using sequential adaptation (Chary et al., 2022). The assessment of the morphology, functionality, and genotype of A549 cells in the different media was performed, uncovering media-specific effects. One of the media formulations results in reduced growth rate, heterogenous cell sizes, differential gene expression, and increased sensitivity to toxicants, suggesting differentiation into alveolar type I and type II epithelial cell phenotypes. Thus, in this medium, cellular differentiation more closely resembles the *in vivo* situation, whereas the other media formulation primarily supported proliferation and maintained a phenotype and morphology similar to that found for FBS-cultured cells. These studies rely on commercially available media, whose exact composition is not fully disclosed, making it difficult or even impossible to determine whether specific components influence observed cellular behaviors, phenotypic changes, or any other experimental outcomes (Chary et al., 2022). Therefore, researchers are increasingly advocating for open science and the use of non-proprietary media resulting in a global effort to develop defined FBS-free media. As an example, Oredsson universal replacement medium (OUR medium) is designed to be a universal medium for 2D and 3D culture (Rafnsdóttir et al., 2023; Oredsson et al., 2024; Malakpour-Permlid et al., 2025b), accompanied by detailed open access production protocols (Weber et al., 2024; Oredsson et al., 2025). The effectiveness of OUR medium was demonstrated through growth and dose-response curves of cells grown in 2D and 3D cultures, along with applications such as cell migration studies (Rafnsdóttir et al., 2023). So far, 23 different cell lines have been adapted to the medium like the A549 and THP-1 cells presented above (Malakpour-Permlid et al., 2025b; Oredsson et al., 2025), as well as spheroids from primary human hepatocytes (Mickols, 2025; Mickols et al., 2025). Further characterization is needed for comparison with the data of Chary et al. (2022) to investigate media specific effects.

As previously mentioned, FBS can induce changes in gene expression. This was visualized in a study by Bieback et al. (2010): A transcriptional analysis revealed 102 genes differentially expressed in ASCs cultured with FBS *versus* human serum or thrombin-activated platelet release plasma. Hence, transitioning from an FBS-containing to an FBS-free medium can favor specific phenotypes, potentially leading to the selection of distinct subclones within a cell population. This highlights the importance of monitoring cellular behavior during the adaptation process, as different clones may emerge even within the same medium type (Cooper et al., 2016; Tièche et al., 2019; Chary et al., 2022).

## 4 Challenges of FBS replacement

This review presents the various efforts to replace FBS and highlights the challenges that explain why the process is sometimes not straightforward. Many FBS-free media still contain animal-derived products, often serum proteins, thus mirroring the reproducibility, safety, and ethical disadvantages of FBS.

### 4.1 Hidden animal-derived materials

A prominent example is albumin, the principal protein component in human blood (Ancell, 1839; Carter and Ho, 1994). In practice, bovine serum albumin (BSA) is frequently used in cell culture media (Pfeifer et al., 2024), even though bovine-derived proteins can contain substances xenogenic to humans, such as *N-*glycolylneuraminic acid (Neu5Gc), potentially compromising the safety of therapeutic cellular products (Rohrer et al., 1998; Heiskanen et al., 2007; Nystedt et al., 2010; Hutton et al., 2024). In many cases, it is not well-communicated that animal-derived proteins like BSA are part of a laboratory material, such as in the supplement B27 (Yao et al., 2006; Chen et al., 2008), unless explicitly stated as xeno-free B27 (Lukovic et al., 2017; Martin E.-R. et al., 2022). In the following example, BSA is hidden behind two layers of trade names: Classically, KnockOut™ Serum Replacement (KOSR or KSR) has been commonly used for adaptation, culturing, passaging, and cryopreservation (Wagner and Welch, 2010a; Wagner and Welch, 2010b) as well as improving the reprogramming efficacy of stem cells (Zhao et al., 2010). However, one of its components is AlbuMAX®, which is lipid-rich BSA (Cranmer et al., 1997; Zhang and Robinson, 2005; Garcia-Gonzalo and Izpisúa Belmonte, 2008; Ogawa et al., 2024), making KOSR problematic for culturing cells for clinical use according to Ludwig and Thomson (2007). Moreover, Martin et al. (2005) identified KOSR as a major source of the xenoantigen Neu5Gc. This illustrates that finding truly animal-component-free media always requires a peek (or sometimes a deep dive) into the specific formulations and details of supplements and media to ensure they are truly free of animal-derived materials. Rivera-Ordaz et al. (2021) state that “formulations of commercially available media remain largely undisclosed, even though reference to the original published compositions is sometimes stated by the manufacturer”, with notable exception like the fully disclosed formulation of Advanced DMEM/F12, which also contains AlbuMAX® (Thermo Fisher Scientific, 2025b). Therefore, open access databases are invaluable tools (see Table 6), but do not relieve scientists from using their critical mind. Consequently, the use of HSA and other human proteins is recommended for human-cell-based biotechnological applications over their bovine counterparts (Penhallow et al., 1986; Maier et al., 2021; Mishra and Heath, 2021). Furthermore, research into albumin-free cell culture, e.g., Chen G. et al. (2011) showed how to cultivate human ESCs and iPSCs in a medium devoid of albumin.

**TABLE 6:** List of databases for animal-free materials (Lima et al., 2020; 3Rs Centre Utrecht, 2024; 3Rs Centre Utrecht, 2025; Modi, 2024).

Database Name	Provider	Links
Basement Membrane Extract (BME)-free Database	3Rs Centre Utrecht	https://bme-free.sites.uu.nl/
Fetal Calf Serum (FCS)-free Database	3Rs Centre Utrecht	https://fcs-free.sites.uu.nl/ https://fcs-free.org/
AntiBodies Chemically Defined (ABCD) Database	Geneva Antibody Facility	https://web.expasy.org/abcd/
Recombinant Antibodies and Mimetics Database	Centre for Human Specific Research	https://antibodies.humanspecificresearch.org/

### 4.2 Animal-derived “replacements”

Further approaches to “replace” FBS with different problematic animal-derived materials include egg white extract (Lee et al., 2024), bovine ocular fluid, earthworm heat-inactivated coelomic fluid, invertebrate fluids, non-bovine sera (Subbiahanadar Chelladurai et al., 2021), and animal tissue hydrolysates (Schlaeger, 1996; Siemensma et al., 2008). Even porcine or bovine platelet lysate is considered (Johansson et al., 2003; Hahn et al., 2024). Others supplement medium containing FBS with additional animal-derived materials, such as extracts from shrimp ovaries and eye stacks (Zhao and Guo, 2023). However, all these examples will just reintroduce the mentioned disadvantages of animal-derived materials back into science, while just shifting ethical issues from bovine fetuses to other animals. Another example is sericin, a globular protein with adhesive and gelatin-like characteristics found in the cocoons of the domestic silk moth (*Bombyx mori*) (Zhang, 2002). While sericin has been shown to have positive effects on cell attachment, proliferation, and survival rates (Tsubouchi et al., 2005; Yanagihara et al., 2006; Liu et al., 2016), its production requires the systematic killing of large numbers of animals, making the use of sericin unethical. Thus, obtaining sericin recombinantly or identifying structurally and functionally similar proteins from plant, fungal, or microbial sources opens up animal-free possibilities.

### 4.3 Algae- and plant-derived materials

Other options include materials derived from algae or plants, like native proteins from the mixotrophically cultured red algae *Galdieria sulphuraria* (Eisenberg et al., 2025), soy hydrolysates (Mattick et al., 2015), and plant-based agro-industrial wastes (Teng et al., 2023; Flaibam et al., 2024b). They contain amino acids, vitamins, and lipids essential for cell culture (Ho et al., 2021). While their limitations, such as lack of standardization and the presence of potential plant-based anti-nutrients, do not yet justify their endorsement for use in scientific research, they may still represent an option for more accessible and sustainable cultured meat production (Etemadian et al., 2021; Flaibam et al., 2024a; Eisenberg et al., 2025). Nevertheless, due to the complexity of hydrolysates, it may be necessary to conduct proteomics and peptidomics analyses to assess their quality and gather information on potential allergens (Carrasco-Castilla et al., 2012).

### 4.4 Xeno-free is key

In general, if proteins are necessary for maintaining the functionality of cells in culture, chemically defined cell culture media with recombinant proteins are the optimal choice to reduce variability and minimize contamination risks.

Ultimately, a xeno-free approach in cell culture should be every scientist’s aim, as working with xenogenic materials creates a chimeric environment. For example, culturing simian cells with bovine serum on a murine matrix, detached with a crustacean-derived dissociation agent (Luce et al., 2022) would generate experimental results with high variability and questionable transferability toward human biology. Science must move away from using animal-derived materials, just like it is moving away from using live animals for scientific purposes.

## 5 Discussion

In recent years, significant advancements have been made in developing serum-free and chemically defined media to support long-term stable cell culturing. Various strategies have been proposed and adopted, including the use of supplementary recombinant proteins (e.g., rHSA, rBSA), hPL, non-animal (i.e., plants, fungi, yeast) extracts or hydrolysates, and chemically defined media (O’Neill et al., 2021; Hanyu et al., 2023). Proteins like growth factors, attachment factors, and hormones are critical for an effective serum replacement, serving as essential components of cell culture media and remain by far the most expensive components in current formulations of serum-free media (Specht, 2020). Notably, innovative methods for the recombinant production of proteins have been developed, with focus on cost reduction (Venkatesan et al., 2022; Mainali et al., 2025) and enhancing expression efficiency (Bobik et al., 2019; Zhu et al., 2021; Maity et al., 2022; Lei et al., 2023). However, using recombinant proteins is not a guarantee for instant success in media development (Jožef et al., 2025). If possible, species-specific recombinant proteins are preferred. Mogilever et al. (2025) used recombinant human and murine proteins in their animal-component-free medium formulation to culture cells of human and murine origin. This can serve as a starting point to customize media components according to the species origin of the utilized cells.

**FIGURE 2:**
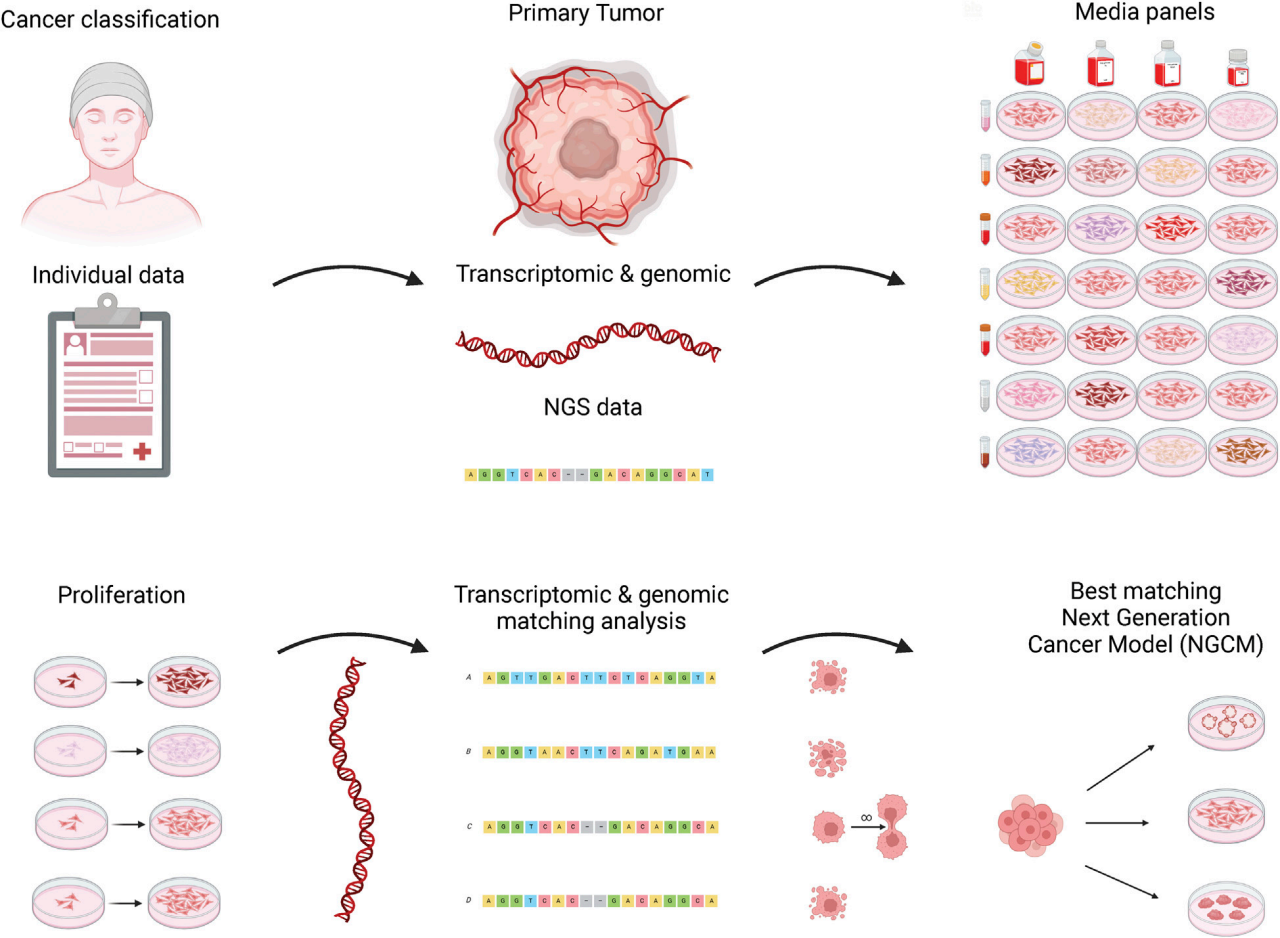
Scheme for the establishment of next-generation cancer models (NGCMs). Top row from left to right: A patient suffering from cancer donates a biopsy sample together with personal medical data obtained in the hospital. In the next step, genomic and transcriptomic molecular data of the primary cells are generated, and a panel of combinations of chemically defined culture media and additives is used to induce proliferation. Bottom row from left to right: Once the optimal medium has been found, a transcriptome is generated again of growing cell cultures and compared with the next-generation sequencing (NGS) data set of the original tumor. Cell lines with the best match are made available to cancer researchers around the world for research into cancer biology and the discovery of new drug targets. The Human Cancer Models Initiative has developed over 700 NGCMs to convert patient tumor samples into a cell model without using fetal bovine serum (FBS). Created in BioRender (https://biorender.com/).

Culturing cells in a defined and transparent environment will *ipso facto* enable scientists to not just reliably compare the results of their experiments across different labs but also facilitate understanding how cells behave *in vitro,* instead of operating in the dark field of the unknown and undefined media. This approach is especially important in applied research and therapeutic medicine. In particular, fields such as translational cancer research, cell-based therapy, and regenerative medicine, are increasingly focusing on *ex vivo* cell culture. To improve cancer research, the Human Cancer Models Initiative of the USA’s National Cancer Institute was founded in 2015 as an international consortium to develop novel and FBS-free next-generation cancer models (NGCMs) based on genomic, clinical, and biospecimen data (see Figure 2). Using chemically defined media for conditional immortalization, the NGCMs provide a unique opportunity for the scientific community to study individual human tumors *in vitro.* This initiative will contribute to the advancement of knowledge in a variety of research areas, such as the development of new cancer therapeutics, cancer biology, biochemistry, and genetics, to determine mechanisms of drug resistance and to assess response to small molecules.

From a political perspective, the transition toward animal-free materials could be supported by expanding Directive 2010/63/EU on the use of animals in scientific procedures (European Union, 2010). This Directive states that member states of the EU “shall ensure that, wherever possible, a scientifically satisfactory method or testing strategy, not entailing the use of live animals, shall be used instead of a procedure.” Since this Directive also applies to fetal forms of mammals from the last third of their normal development, obtaining blood from those fetuses for FBS production would require a project evaluation and proper cost-benefit analysis for a procedure under the Directive (van der Valk et al., 2018). Unfortunately, this still leaves open several loopholes, including the use of less developed fetuses or those that died before the start of the procedure. Nonetheless, the production of other animal-derived materials, such as Matrigel®, undoubtedly involves live animals under the scope of the Directive. Therefore, the existence of animal-free replacements should prevent such procedures, at least within the EU under Directive 2010/63/EU. A subsequent step would be to regulate that, as soon as it is possible to use an animal-free material, the corresponding animal-derived material must no longer be used in scientific procedures. Complementing this, projects for the development and validation of animal-free materials, as well as funding opportunities, should be created. Initiatives like the “non-animal derived product validation awards” can serve as a role model for governments and 3Rs centers around the globe (NC3Rs, 2024).

Scientists can proactively state the ethical implications when using animal-derived material in their studies (Malakpour-Permlid et al., 2025a). A big push toward a paradigm shift would be that journals and reviewers demand animal-free materials for *in vitro* publications and a clear scientific justification when animal-derived materials are used (Weber et al., 2022). The journal ALTEX (2024) antecedes by recommending “the substitution of all materials that are obtained or derived from animals subjected to pain or suffering” for submitted manuscripts, and if the authors have used such materials it is required “to discuss this issue, preferably in the Discussion section, and indicate whether such materials could/shall be replaced in future studies.” This practice is recommended, and authors, reviewers, and editors are encouraged to adopt it.

Trust is good, but transparency is better. Likewise, manufacturers and regulators can do their part by labeling cell culture products as free from animal-derived materials. In addition, a variability score for product components would contribute significantly to scientific reproducibility. Furthermore, cell line providers should also remove animal-derived media from their recommended culture conditions, for example by using the information in this publication.

Fortunately, an increasing number of FBS-free cell culture models have arrived on the market and in scientific literature. Unfortunately, if the routine culturing of cells continues to be done in FBS, the gain from achieving an FBS-free culture model is relatively minuscule. Therefore, the goal should always be a full replacement of animal-derived materials throughout the whole process of cell culturing, starting from the source to the cell bank, and extending from the bench to the bedside.

**FIGURE 3:**
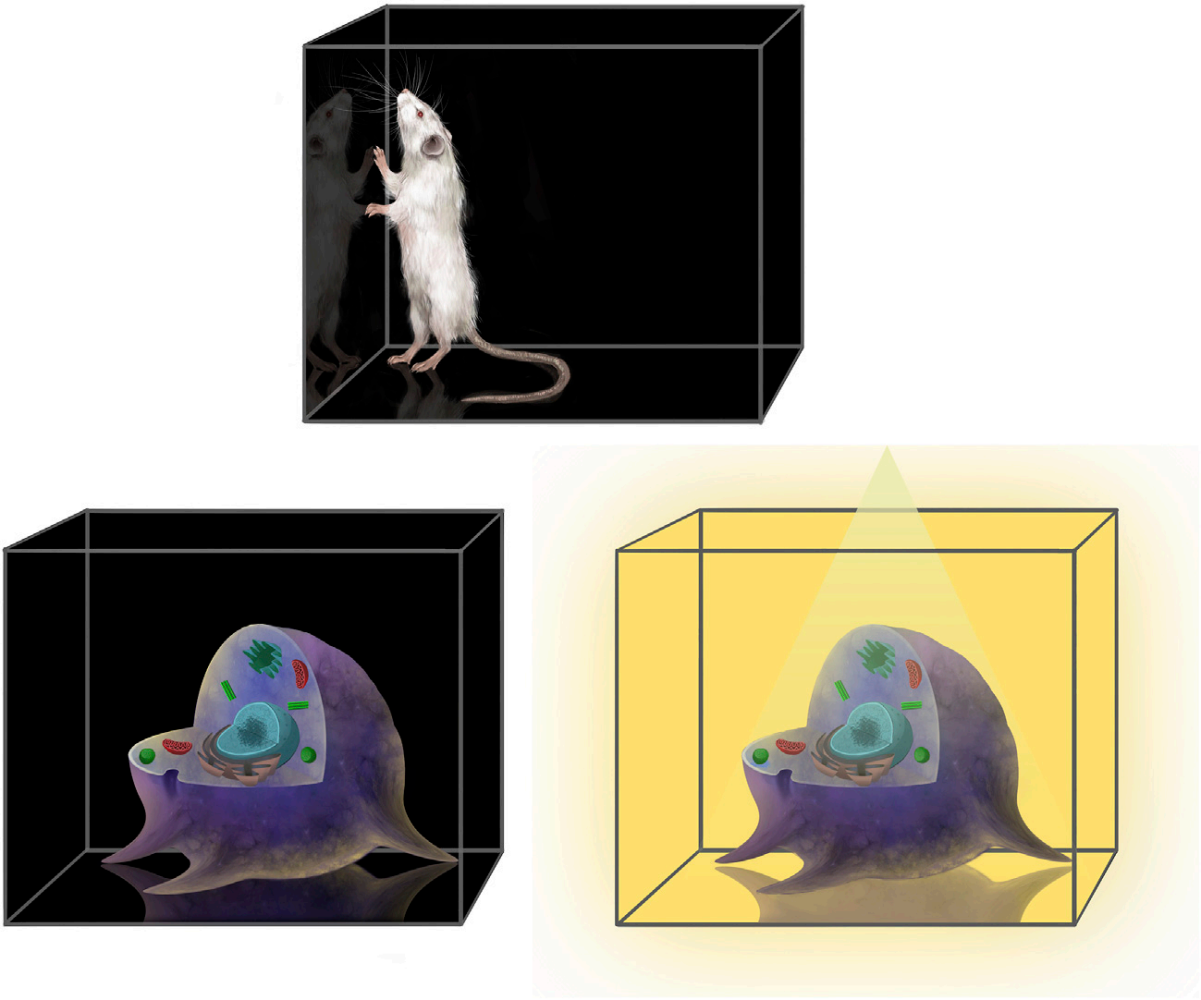
Lighting up the black box of cell culture experiments. An animal experiment is a black box, as well as culturing cells in a medium with unknown composition. Using a medium with disclosed composition will illuminate the black box and improve cell culture research. Created by Kristina Kostova (https://www.kriskostova.com/).

An animal experiment is a black box (Calabrese, 1987), due to issues of transferability, translatability, artificiality, and variability. In contrast, experimenting *ex vivo* on cells from the species of interest represents a giant leap toward more transferable and translatable results (Ban et al., 2020). However, as long as the medium of the cells is proprietary and/or undefined, artificiality and variability will persist, thereby keeping the cells in a black box as well. Yet given the availability of media with disclosed compositions, this black box can be illuminated (see Figure 3). Only then can science focus on studying the important question: What is happening in the cell?

## 6 Conclusion

Science thrives on change: Scientific knowledge constantly grows, while life science research, methods, and models are constantly adjusting and evolving accordingly. This should also be the case for presumed long-standing gold standards and state-of-the-art methods. Scientists and other stakeholders have demonstrated successful rethinking of gold standards in the past. For instance, the rabbit pyrogen test to detect fever-inducing toxins in medical products was developed in 1912 (Hort and Penfold, 1912). In 2024, it was decided to officially omit the rabbit pyrogen test from the European Pharmacopoeia as of January 2026 (EDQM, 2024; Council of Europe, 2025), given that an animal-free method had been demonstrated to be a suitable replacement for the animal test (Hoffmann et al., 2005; Cirefice et al., 2023). Just like the replacement of the rabbit pyrogen test, which might have seemed impossible in the past, the replacement of FBS is in progress and will gradually make it dispensable.

In summary, this review highlights a range of animal-free materials that can potentially replace FBS in research across biological, medical, pharmaceutical sciences, and food industries. It clearly demonstrates that it is possible to leave FBS (and other animal-derived materials) behind and pursue a more reliable and ethical science.

## Nomenclature

ASCs: adipose tissue-derived MSCs
BMSCs: bone marrow-derived MSCs
BSA: bovine serum albumin
BSCs: bovine satellite cells
CAR T: chimeric antigen receptor T
cGMP or GMP: current good manufacturing practice
CHO: Chinese hamster ovary
CIVMs: complex *in vitro* models
DMSO: dimethyl sulfoxide
ESCs: embryonic stem cells
EU: European Union
FBS: fetal bovine serum
FCS: fetal calf serum
FDA: Food and Drug Administration
h-CLAT: human cell line activation test
HEK293: human embryonic kidney 293
hPL: human platelet lysate
HSA: human serum albumin
HUVECs: human umbilical vein endothelial cells
iPSCs: induced pluripotent stem cells
ISIA: International Serum Industry Association
ISO: International Organization for Standardization
KOSR or KSR: KnockOut™ Serum Replacement
MDCK: Madin-Darby canine kidney
MPS: microphysiological systems
MSCs: mesenchymal stem/stromal cells
Neu5Gc: *N*-glycolylneuraminic acid
NGCMs: next-generation cancer models
OECD: Organisation for Economic Co-operation and Development
OoC: organ-on-a-chip
OUR: Oredsson universal replacement
PBS: phosphate-buffered salt
rBSA: recombinant bovine serum albumin
rHSA: recombinant human serum albumin
Sf9: *Spodoptera frugiperda* 9
TG: test guidelines
USA: United States of America
