# Supplementary material for: Fetal bovine serum: how to leave it behind in the pursuit of more reliable science
Source: Front Toxicol. 2025 Aug 8;7:1612903. doi: 10.3389/ftox.2025.1612903 (PMC12371577; doi:10.3389/ftox.2025.1612903)
Supplement: Supplementary file 1 [file Table1.pdf]

Supplementary Material for doi: 10.3389/ftox.2025.1612903

Supplementary Table 1: Cell types successfully cultured and supported by a serum-free medium on an organ-on-a-chip (OoC) system.

**Supported Cells**	**References**
Adult hippocampal neurons	Varghese et al. (2009); Edwards et al. (2010)
Motoneurons	Das et al. (2003, 2007a); Guo et al. (2010, 2014, 2017, 2020)
Sensory neurons	Rumsey et al. (2010); Guo et al. (2013)
Neuromuscular junction formation cells	Guo et al. (2011, 2020); Smith et al. (2013)
Cardiac cells	Das et al. (2004); Natarajan et al. (2006, 2011); Stancescu et al. (2015)
Muscle cells	Das et al. (2006, 2007b, 2009a, 2009b); Badu-Mensah et al. (2020)
Liver cells	Oleaga et al. (2016, 2021)
Endothelial cells	Rolland et al. (2008)
Epithelial cells	Rolland et al. (2008)

References

Badu-Mensah, A., Guo, X., McAleer, C. W., Rumsey, J. W., and Hickman, J. J. (2020). Functional skeletal muscle model derived from SOD1-mutant ALS patient iPSCs recapitulates hallmarks of disease progression. *Sci. Rep.* 10, 14302. doi: 10.1038/s41598-020-70510-3

Das, M., Gregory, C. A., Molnar, P., Riedel, L. M., Wilson, K., and Hickman, J. J. (2006). A defined system to allow skeletal muscle differentiation and subsequent integration with silicon microstructures. *Biomaterials* 27, 4374–4380. doi: 10.1016/j.biomaterials.2006.03.046

Das, M., Molnar, P., Devaraj, H., Poeta, M., and Hickman, J. J. (2003). Electrophysiological and morphological characterization of rat embryonic motoneurons in a defined system. *Biotechnol. Prog.* 19, 1756–1761. doi: 10.1021/bp034076l

Das, M., Molnar, P., Gregory, C., Riedel, L., Jamshidi, A., and Hickman, J. J. (2004). Long-term culture of embryonic rat cardiomyocytes on an organosilane surface in a serum-free medium. *Biomaterials* 25, 5643–5647. doi: 10.1016/j.biomaterials.2004.01.020

Das, M., Rumsey, J. W., Bhargava, N., Gregory, C., Reidel, L., Kang, J. F., et al. (2009a). Developing a novel serum-free cell culture model of skeletal muscle differentiation by systematically studying the role of different growth factors in myotube formation. *Vitro Cell. Dev. Biol. - Anim.* 45, 378–387. doi: 10.1007/s11626-009-9192-7

Das, M., Rumsey, J. W., Bhargava, N., Stancescu, M., and Hickman, J. J. (2009b). Skeletal muscle tissue engineering: A maturation model promoting long-term survival of myotubes, structural development of the excitation-contraction coupling apparatus and neonatal myosin heavy chain expression. *Biomaterials* 30, 5392–5402. doi: 10.1016/j.biomaterials.2009.05.081

Das, M., Rumsey, J. W., Gregory, C. A., Bhargava, N., Kang, J.-F., Molnar, P., et al. (2007a). Embryonic motoneuron-skeletal muscle co-culture in a defined system. *Neuroscience* 146, 481–488. doi: 10.1016/j.neuroscience.2007.01.068

Das, M., Wilson, K., Molnar, P., and Hickman, J. J. (2007b). Differentiation of skeletal muscle and integration of myotubes with silicon microstructures using serum-free medium and a synthetic silane substrate. *Nat. Protoc.* 2, 1795–1801. doi: 10.1038/nprot.2007.229

Edwards, D., Das, M., Molnar, P., and Hickman, J. J. (2010). Addition of glutamate to serum-free culture promotes recovery of electrical activity in adult hippocampal neurons *in vitro*. *J. Neurosci. Methods* 190, 155–163. doi: 10.1016/j.jneumeth.2010.04.030

Guo, X., Badu-Mensah, A., Thomas, M. C., McAleer, C. W., and Hickman, J. J. (2020). Characterization of functional human skeletal myotubes and neuromuscular junction derived—from the same induced pluripotent stem cell source. *Bioengineering* 7, 133. doi: 10.3390/bioengineering7040133

Guo, X., Colon, A., Akanda, N., Spradling, S., Stancescu, M., Martin, C., et al. (2017). Tissue engineering the mechanosensory circuit of the stretch reflex arc with human stem cells: Sensory neuron innervation of intrafusal muscle fibers. *Biomaterials* 122, 179–187. doi: 10.1016/j.biomaterials.2017.01.005

Guo, X., Das, M., Rumsey, J., Gonzalez, M., Stancescu, M., and Hickman, J. J. (2010). Neuromuscular junction formation between human stem-cell-derived motoneurons and rat skeletal muscle in a defined system. *Tissue Eng. Part C Methods* 16, 1347–1355. doi: 10.1089/ten.TEC.2010.0040

Guo, X., Gonzalez, M., Stancescu, M., Vandenburgh, H. H., and Hickman, J. J. (2011). Neuromuscular junction formation between human stem cell-derived motoneurons and human skeletal muscle in a defined system. *Biomaterials* 32, 9602–9611. doi: 10.1016/j.biomaterials.2011.09.014

Guo, X., Greene, K., Akanda, N., Smith, A., Stancescu, M., Lambert, S., et al. (2014). *In vitro* differentiation of functional human skeletal myotubes in a defined system. *Biomater. Sci.* 2, 131–138. doi: 10.1039/C3BM60166H

Guo, X., Spradling, S., Stancescu, M., Lambert, S., and Hickman, J. J. (2013). Derivation of sensory neurons and neural crest stem cells from human neural progenitor hNP1. *Biomaterials* 34, 4418–4427. doi: 10.1016/j.biomaterials.2013.02.061

Natarajan, A., Molnar, P., Sieverdes, K., Jamshidi, A., and Hickman, J. J. (2006). Microelectrode array recordings of cardiac action potentials as a high throughput method to evaluate pesticide toxicity. *Toxicol. In Vitro* 20, 375–381. doi: 10.1016/j.tiv.2005.08.014

Natarajan, A., Stancescu, M., Dhir, V., Armstrong, C., Sommerhage, F., Hickman, J. J., et al. (2011). Patterned cardiomyocytes on microelectrode arrays as a functional, high information content drug screening platform. *Biomaterials* 32, 4267–4274. doi: 10.1016/j.biomaterials.2010.12.022

Oleaga, C., Bernabini, C., Smith, A. S. T., Srinivasan, B., Jackson, M., McLamb, W., et al. (2016). Multi-organ toxicity demonstration in a functional human *in vitro* system composed of four organs. *Sci. Rep.* 6, 20030. doi: 10.1038/srep20030

Oleaga, C., Bridges, L. R., Persaud, K., McAleer, C. W., Long, C. J., and Hickman, J. J. (2021). A functional long-term 2D serum-free human hepatic *in vitro* system for drug evaluation. *Biotechnol. Prog.* 37, e3069. doi: 10.1002/btpr.3069

Rolland, J. P., Lee, K.-S., Mahmood, A., Fluck, L., Duarte, J., Kaya, I., et al. (2008). "Collaborative engineering: 3-D optical imaging and gas exchange simulation of *in-vitro* alveolar constructs," in *Medicine meets virtual reality 16*, eds. J. D. Westwood, R. S. Haluck, H. M. Hoffman, G. T. Mogel, R. Phillips, R. A. Robb, et al. (Amsterdam, The Netherlands: IOS Press), 426–432. Available at: https://ebooks.iospress.nl/publication/11372

Rumsey, J. W., Das, M., Bhalkikar, A., Stancescu, M., and Hickman, J. J. (2010). Tissue engineering the mechanosensory circuit of the stretch reflex arc: Sensory neuron innervation of intrafusal muscle fibers. *Biomaterials* 31, 8218–8227. doi: 10.1016/j.biomaterials.2010.07.027

Smith, A. S. T., Long, C. J., Pirozzi, K., and Hickman, J. J. (2013). A functional system for high-content screening of neuromuscular junctions *in vitro*. *Technol. Singap. World Sci.* 1, 37–48. doi: 10.1142/S2339547813500015

Stancescu, M., Molnar, P., McAleer, C. W., McLamb, W., Long, C. J., Oleaga, C., et al. (2015). A phenotypic *in vitro* model for the main determinants of human whole heart function. *Biomaterials* 60, 20–30. doi: 10.1016/j.biomaterials.2015.04.035

Varghese, K., Das, M., Bhargava, N., Stancescu, M., Molnar, P., Kindy, M. S., et al. (2009). Regeneration and characterization of adult mouse hippocampal neurons in a defined *in vitro* system. *J. Neurosci. Methods* 177, 51–59. doi: 10.1016/j.jneumeth.2008.09.022
